# The Bexsero *Neisseria meningitidis* serogroup B vaccine antigen NHBA is a high-affinity chondroitin sulfate binding protein

**DOI:** 10.1038/s41598-018-24639-x

**Published:** 2018-04-25

**Authors:** Tsitsi D. Mubaiwa, Lauren E. Hartley-Tassell, Evgeny A. Semchenko, Christopher J. Day, Michael P. Jennings, Kate L. Seib

**Affiliations:** Institute for Glycomics, Griffith University, Gold Coast, Queensland Australia

## Abstract

*Neisseria meningitidis* is a Gram-negative bacterial pathogen that causes life threatening meningitis and septicemia. Neisseria Heparin Binding Antigen (NHBA) is an outer membrane protein that binds heparin and heparan sulfate and DNA. This protein is one of the four antigens in the meningococcal serogroup B vaccine Bexsero. In the current study, we sought to define the full glycan-binding repertoire of NHBA to better understand its role in meningococcal pathogenesis and vaccine efficacy. Glycan array analysis revealed binding to 28 structures by recombinant NHBA. Surface plasmon resonance was used to confirm the binding phenotype and to determine the affinity of the interactions. These studies revealed that the highest affinity binding of NHBA was with chondroitin sulfate (K_D_ = 5.2 nM). This affinity is 10-fold higher than observed for heparin. Analysis of binding with well-defined disaccharides of the different chondroitin sulfate types demonstrated that the most preferred ligand has a sulfate at the 2 position of the GlcA/IdoA and 6 position of the GalNAc, which is an equivalent structure to chondroitin sulfate D. Chondroitin sulfate is widely expressed in human tissues, while chondroitin sulfate D is predominantly expressed in the brain and may constitute a new receptor structure for meningococci.

## Introduction

*Neisseria meningitidis* is a Gram-negative bacterial pathogen that causes life threatening meningitis and septicemia^1^. The organism is susceptible to antibiotics; however, it is difficult to diagnose at early stages and can rapidly progress to a life-threatening disease. The combination of difficult diagnosis and rapid progression indicates that vaccination is the most effective and appropriate public health response to this organism. There are 13 serogroups of *N. meningitidis* based on the expression of different polysaccharide capsule structures. Serogroup A is the dominant serogroup in sub-saharan Africa, where the highest burden of disease exists^2^. Serogroups B and C predominate in the developed world^3^, although serogroups X and W-135 are also emerging as a major cause of disease in several regions worldwide^2,3^. The polysaccharide capsules that are the basis of these serogroups have been used as antigens in highly successful conjugate vaccines, including monovalent serogroup A and C vaccines, and four-valent vaccines comprising A, C, W-135 and Y capsule polysaccharides^4^. The same capsule-based vaccine approach cannot be used for serogroup B strains, as the alpha-2-8 polysialic acid expressed by these strains is a structural mimic of human neural cell adhesion molecule (NCAM)^5^ and may induce immunopathology if used as a vaccine antigen. Therefore, serogroup B vaccine development had to move in a different direction, utilizing surface proteins of this highly variable pathogen.

In 2013, a serogroup B vaccine (4CMenB, Bexsero) was licensed for use. This vaccine contains four components; outer membrane vesicles from a serogroup B strain, formulated with three recombinant proteins NadA, fHBP and NHBA^6,7^. These outer membrane proteins provide targets for complement-mediated serum bactericidal activity^6^. They also provide an opportunity for functional blocking as each is proposed to play a key role in *N. meningitidis* pathogenesis, and understanding the functional roles of these surface antigens enables a better understanding of how the vaccine is functioning to protect against infection. NadA is an adhesin^8^ and fHBP binds to human factor H in serum reducing the efficiency of complement mediated killing^9,10^. NHBA binds heparin^11^ and heparan sulfate proteoglycans^12^ via an arginine (Arg)-rich region. Two proteases, the phase variable meningococcal NalP and human lactoferrin have been shown to cleave NHBA upstream and downstream of the central Arg-rich region, respectively^11^. The functional significance of these cleavage events is yet to be elucidated. NHBA binding to heparin mediates increased serum resistance^11^, potentially via interactions between heparin and factor H or C4b binding protein^11,13^. NHBA is also involved in meningococcal adherence to epithelial cells, via binding to heparan sulfate proteoglycans^12^. NHBA also binds to DNA, and although this interaction has not been fully characterized, NHBA-DNA binding promotes biofilm formation^14^. The *nhbA* gene is found in all meningococcal strains tested^7,15^.

The lectin (carbohydrate binding) activity of NHBA for heparin and heparin sulfate has been well characterized and is believed to be important for meningococcal pathogenesis. In the current study, we sought to investigate the full range of lectin activity of NHBA using glycan array analysis.

## Results

### Glycan array analysis reveals additional lectin activity of NHBA

**Figure 1:**
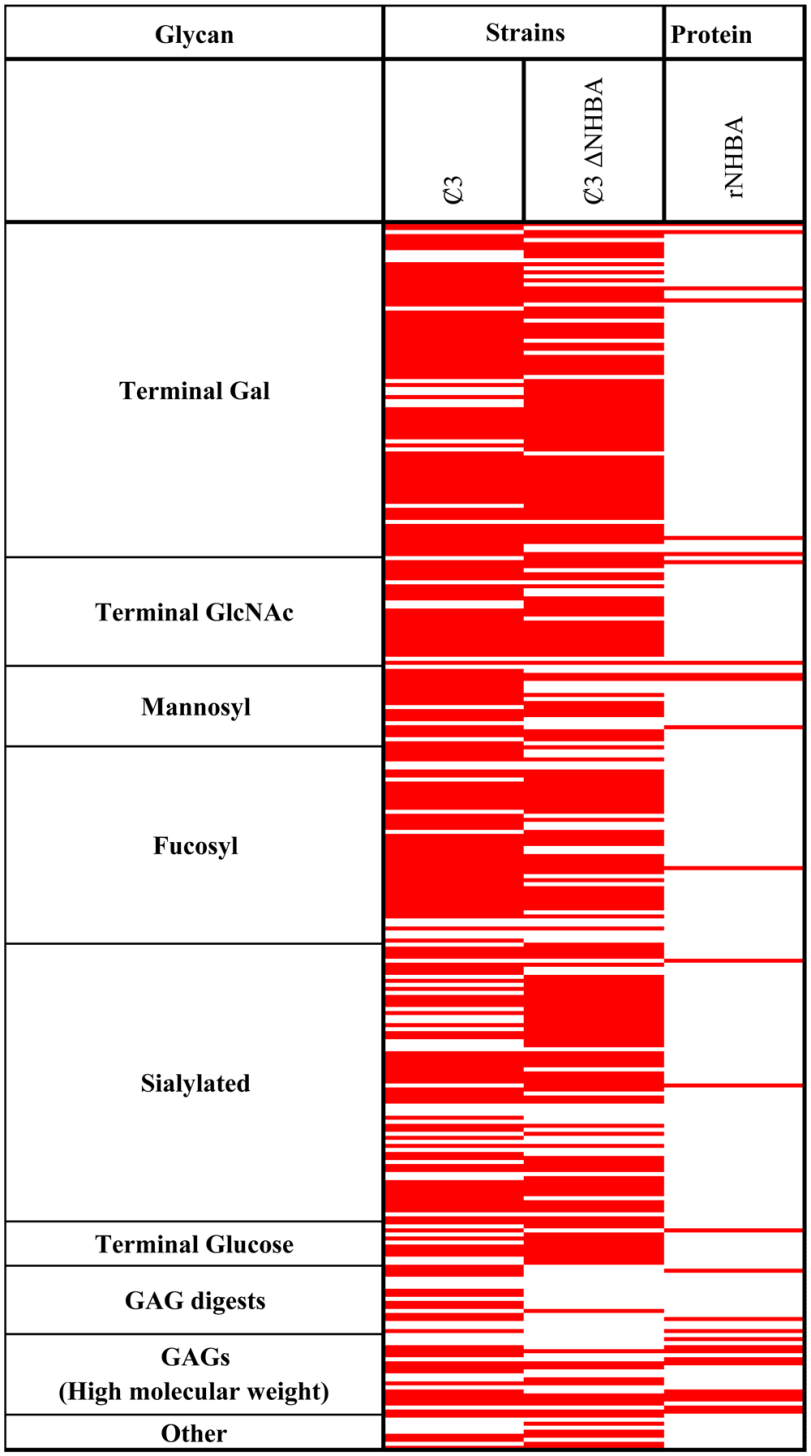
Heat map showing glycan binding by NHBA. The glycan binding properties of unencapsulated *N. meningitidis* (Ȼ3) are compared to that of an isogenic NHBA mutant (Ȼ3ΔNHBA) and recombinant NHBA (rNHBA). Red represents binding (in three independent replicates) and white is no binding observed.

To determine the lectin activity of NHBA we performed glycan array analysis using an unencapsulated MC58 strain (Ȼ3) in comparison with an isogenic *nhbA* mutant strain (Ȼ3Δ*nhbA*). Fluorescently labelled bacteria were incubated on the Institute for Glycomics’ v3.0 glycan array^16^. The results shown in Fig. 1 (see also Supplementary Table S1) reveal that strain Ȼ3 binds to 61 structures that the Ȼ3Δ*nhbA* strain does not bind. Direct binding to glycans by NHBA was also investigated using purified recombinant NHBA, which revealed binding to 28 glycan structures on the array. Twenty-two of these structures were in common with those bound by the NHBA expressing strain. Seven structures bound both the recombinant NHBA and the wild type strain (but not the Ȼ3Δ*nhbA* strain) indicating NHBA-specific and -dependent binding (see Supplementary Table S1; Index 5D, 46, 12B, 13B, 13E,13J, 14F). These data suggest that the binding observed by the recombinant protein is mainly consistent with that mediated by the protein expressed in its native state on the surface of *N. meningitidis*. However, some glycans that bound the whole cell bacteria in an NHBA-dependent manner did not bind recombinant NHBA, possibly due to the need for native folding of NHBA on the cell surface for binding to occur. Also, several of the recombinant NHBA-binding glycan structure were bound by both the wild type and the *nhbA* mutant strain, suggesting that more than one meningococcal lectin is involved in binding. In addition, some of the recombinant NHBA-binding glycan structures were not bound by the wild type, possibly due to interactions being blocked on the bacterial surface by the presence of other meningococcal components, such as lipooligosaccharide (LOS)^17,18^ or pili^18,19^ that can negatively affect interactions mediated by other structures. The observed NHBA-dependent lectin activity includes the glycosaminoglycans (GAGs) heparin, heparan sulfate, heparinase III digests, hyaluronan, chondroitin sulfate, as well as glucose and the ganglioside GT3 (Fig. 1, Supplementary Table S1).

### Surface Plasmon Resonance analysis of NHBA lectin activity

**Figure 2:**
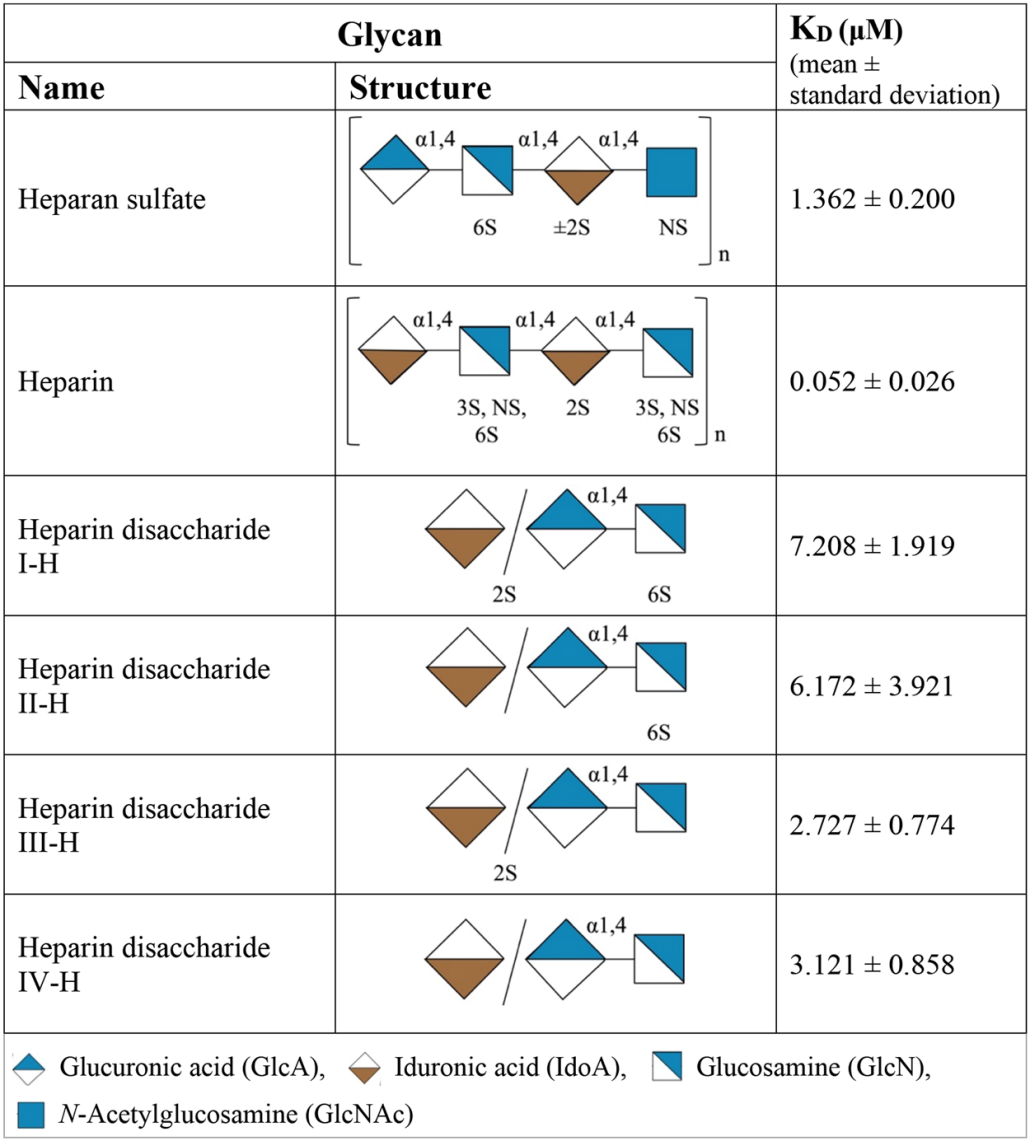
SPR analysis of NHBA interactions with heparan sulfate, heparin and heparin disaccharides. Heparin and heparan sulfate polymers display complex, non-uniform sulfation patterns. The structures provided are representative of the major repeat units. #S: Sulfation at the # carbon. NS: No-sulfation on the specified monomer.

To determine the affinity of NHBA interactions discovered in glycan array analysis, we used surface plasmon resonance (SPR). In these studies, recombinant NHBA was bound covalently to a biosensor chip and various glycans were analyzed in flow for binding to NHBA (see Table 1; Supplementary data S1). The glycans tested by SPR included those that bound to glycan arrays in an NHBA-specific and -dependent manner on whole cells (i.e., bound the wild type but not the *nhba* mutant strain) and that either also bound recombinant NHBA (Table 1; Index 46, 12B, 13E, 13J) or that did not bind recombinant NHBA (Table 1; Index 2E, 8A, 12C). In addition, glycans were tested that bound to recombinant NHBA and that either bound both the wild type and mutant strains (Index 13K), did not bind either the wild type or mutant strains (Index 14J), or that bound the mutant but not the wild type strain (Index 532). NHBA can recognize multiple GAGs, and the highest affinity interaction observed was between NHBA and chondroitin sulfate with a dissociation constant(K_D_) of 5.2 nM. NHBA was named based on its interaction with heparin^11^, and binding to heparan sulfate has subsequently been described^12^. The affinity of NHBA with heparin (13J) was K_D_ = 52 nM, and its affinity with heparan sulfate (14J) was K_D_ = 1.4 µM. Heparin is more sulfated than heparan sulfate^20^, and these data suggest that the sulfation of heparin may be required for high affinity binding by NHBA. We conducted studies with disaccharides representing all possible sulfations of heparin. However, all of these disaccharides showed low affinity binding to NHBA, indicating that NHBA requires a larger binding epitope for the higher affinity interaction observed with the heparin polymer structure (Fig. 2, Supplementary data S1). The unsulfated GAG, hyaluronin, is bound by NHBA with affinity of K_D_ = 130 nM, and binding was also observed to the ganglioside GT3 (K_D_ = 210 nM; Table 1).

**Table 1:** SPR analysis of NHBA-glycan interactions.

Glycan			K_D_ (μM) (mean ± standard deviation)
Index	Name	Structure	
46	β-Glc6P	6-H_2_PO_3_Glcβ-sp4	0.056 ± 0.025
2E	P1 antigen	Galα1-4Galβ1-4GlcNAc	14.93 ± 3.48
8A	Sulfo Lewis A	SO_3_-3Galβ1-3(Fucα1-4) GlcNAc	33.4 ± 2.54
12B	Neocarratetraose-4^1^-O-sulfate (Na+)	C_24_H_37_O_22_SNa	0.44613 ± 0.1511
12C	Neocarrahexaose-2^4^,4^1,3,5^-tetra-O-sulfate (Na+)	C_36_H_52_O_40_S_4_Na_4_	NCDB
13E	Hyaluronan disaccharide ΔDi-HA	GlcA/IdoA -GlcNAc	0.13 ± 0.03
17M	GT3 ganglioside sugar	Neu5Acα2-8Neu5Acβ2-8Neu5Acα2-3Galβ1-4Glc	0.21 ± 0.056
13K	Chondroitin sulfate	(GlcA/IdoAβ1-3(±4/6S) GalNAcβ1-4) n (n < 250)	0.0052 ± 0.0024
14J	Heparan sulfate	(GlcA/IdoAα/β1-4GlcNAcα1-4(±NS)) n (n = ~200)	1.362 ± 0.200
13J	Heparin	(GlcA/IdoAα/β1-4GlcNAcα1-4) n (n = ~200)	0.052 ± 0.026

NCDB: No concentration dependent binding observed within the range of the instrument’s detection.

### NHBA-chondroitin sulfate interaction is a high affinity interaction that requires high, mixed sulfation

**Figure 3:**
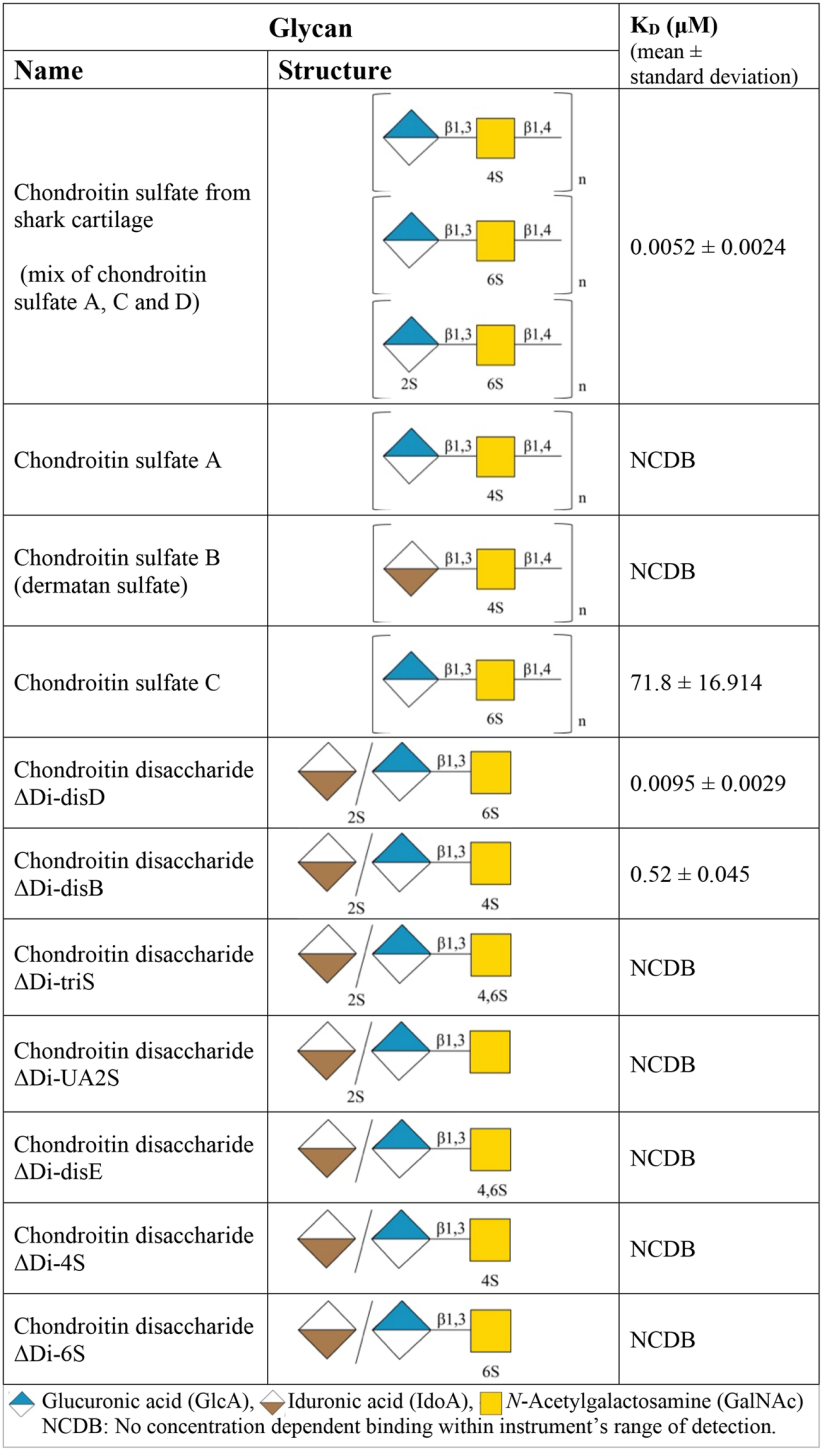
SPR analysis of NHBA-chondroitin interaction. NCDB: No concentration dependent binding observed within the range of the instrument’s detection.

Chondroitin sulfate may have sulfation at different positions on the repeating disaccharide, and the highest affinity interaction observed in our studies was with chondroitin sulfate isolated from shark cartilage (Fig. 3). The manufacturer does not specify the type of chondroitin sulfate in this reagent, however the literature suggests that shark cartilage chondroitin sulfate is a mixture of chondroitin sulfate A, C and D^21–23^. To further characterize the high affinity interaction of NHBA with chondroitin sulfate, we performed SPR studies with a series of chondroitin sulfate polymers and disaccharides with fully characterized structures displaying alternative sulfation patterns (Fig. 3, Supplementary data S1). In terms of the chondroitin sulfate polymers, pure chondroitin sulfate C had 10,000-fold reduction in affinity of binding to NHBA compared to the shark A, C, D mixture. Chondroitin sulfate A and chondroitin sulfate B, showed no binding to NHBA. These studies indicate that NHBA prefers chondroitin sulfate D. Additional studies using chondroitin disaccharides that are either synthesized or purified from a larger polysaccharide confirmed that that highest affinity binding by NHBA requires a sulfate at the 2 position of the GlcA/IdoA and at the 6 position of the GalNAc (K_D_ = 9.5 nM), which is equivalent structure to chondroitin sulfate D. Reduced affinity was observed with a disaccharide that has sulfation at the 2 position of the GlcA/IdoA and 4 position of the GalNAc (K_D_ = 500 nM). No binding was seen if the disaccharide was sulfated at all three positions (2S on GlcA/IdoA and GalNAc with 4S and 6S) or with disaccharides sulfated at a single position (i.e. either 2S on GlcA/IdoA only or GalNAc with 4S only or GalNAc 6S only; Fig. 3). This indicates that the binding of NHBA to chondroitin sulfate is highly specific and that the position of the sulfation affects the affinity of the interaction.

### Competition between chondroitin sulfate, heparan sulfate and heparin for NHBA binding

**Figure 4:**
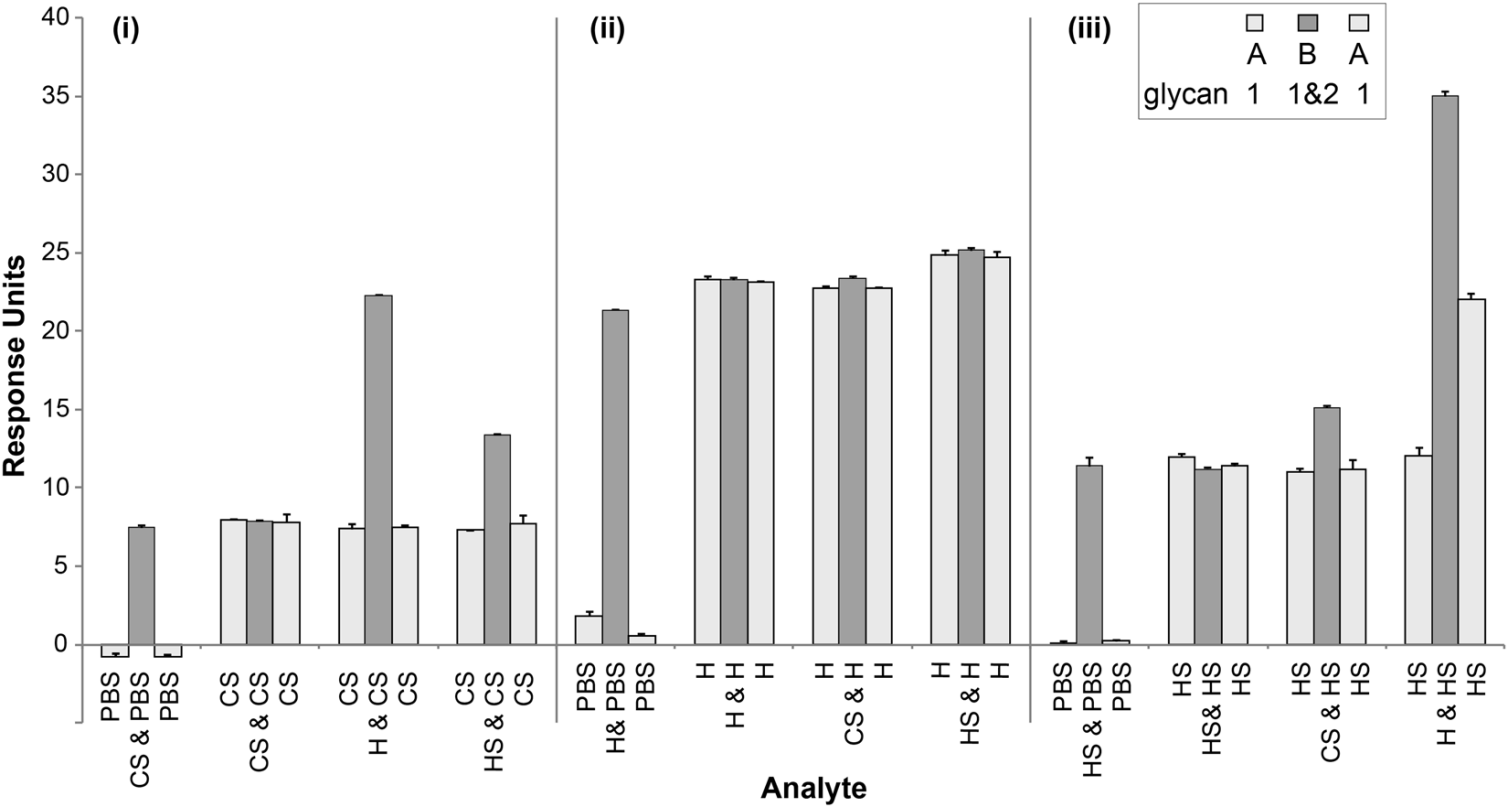
A-B-A SPR competition analysis of binding of glycans to NHBA. Competition of: (**i)** chondroitin sulfate D disaccharide (CS) with heparin (H) or heparan sulfate (HS); (**ii)** H with CS or HS; **(iii)** HS with CS or H. Glycans were used at concentration 10x their respective K_D_s, and injected in the following order: glycan 1 (A) → glycan 1 and glycan 2 (B) → glycan 1 (A). PBS is used as a control.

To investigate the nature of binding sites on NHBA, a competition SPR assay was performed with the three main GAG substrates of NHBA using the A-B-A function of the Biacore S200 software. The assay is designed to show if a cumulative response is observed when a second analyte (B) is flown across the bound protein while the first analyte (A) is present. Analysis of the resultant sensorgrams showed that the binding of chondroitin disaccharide D to NHBA was not able to inhibit the subsequent binding of heparin or heparan sulfate polymers to the NHBA protein, as an additive effect was observed when the disaccharide was added (Fig. 4(i)). When heparin is added first, it inhibits binding of both the chondroitin disaccharide D and heparan sulfate (Fig. 4(ii)). However, heparan sulfate cannot inhibit the binding of either of the chondroitin disaccharide D or the heparin, with additive binding occurring in each case (Fig. 4(iii)). The competition assay suggests that there are two GAG binding sites. The first is a disaccharide specific site, responsible for the high affinity chondroitin sulfate D and heparin binding. The second site is a polymer binding site responsible for the low affinity binding of heparan sulfate, but this region also associates with the high affinity GAG polymers.

### Analysis of NHBA-DNA binding

NHBA has been reported to bind to DNA^14^ so this was further investigated using SPR and ITC. SPR analysis demonstrated interaction between DNA and NHBA with a K_D_ of 0.071 µM (±0.017), but saturation was not reached indicating an interaction that did not have 1:1 stoichiometry (Supplementary data S2). ITC analysis was therefore used, determining the affinity between DNA and NHBA was 3.33 µM (±1.1) with a stoichiometry of 30.73 (±10.89) NHBA bound per 503 bp DNA molecule. This is a relatively low affinity interaction due to the high stoichiometry, suggesting that NHBA has no preference for a specific DNA sequence and is interacting based on the negative charge and the polymeric nature of the DNA.

## Discussion

Glycans are important for host pathogen interactions, and several pathogens that colonize the upper respiratory tract and central nervous system have been shown to target host glycans for their adherence/invasion into host cells^24,25^. Recent work has shown that the meningococcal surface protein NHBA binds to the GAGs heparin and heparan sulfate, and these interactions mediate meningococcal serum resistance^11^ and adherence to host epithelial cells^12^, respectively. In the current study, we sought to fully characterize the lectin activity of NHBA using a comprehensive glycan array, as well as SPR and ITC analysis.

The initial screen using glycan array analysis and the *N. meningitidis* wild type and Δ*nhbA* mutant strains revealed loss of binding to 61 structures by the mutant relative to the wild type, which indicates glycan binding by NHBA. Interestingly, 41 additional interactions on the glycan array were observed with the mutant, that were not seen with the wild type strain. These results suggest a possible inhibitory or modulatory role for NHBA, one similar to that reported for meningococcal antigens such as lipooligosaccharide (LOS)^17,18^, capsule^17,18^ and pili^18,19^; which have been shown to negatively affect interactions mediated by other structures.

NHBA lectin activity was confirmed using recombinant NHBA protein. The recombinant NHBA protein bound to 28 structures on the glycan array, and binding to 22 of these structures was also observed by the wild type *N. meningitidis* Ȼ3 using whole fluorescently labelled bacteria. These data indicate that NHBA has a much broader lectin activity than binding to heparin; the ligand binding activity after which it was named. The majority of the newly identified glycans bound by NHBA are sialylated and/or sulfated, which is consistent with the previously described negatively charged ligands of NHBA, which include heparin^11^, heparan sulfate^12^ and DNA^14^. Furthermore, the majority of the glycans to which NHBA bound were GAGs and included chondroitin sulfate polymers and digests as well as hyaluronan.

Heparan sulfate and chondroitin sulfate are found on a range of host cell surfaces and in the extracellular matrix^26^. Heparan sulfate proteoglycans are typically found on epithelial cells, endothelial cells and fibroblasts^27^, while chondroitin sulfate proteoglycans are typically found on lymphocytes^27^, within the central nervous system^28^, and are highly expressed on endothelial cells^27,29^. It is important to note that the expression of different chondroitin sulfate types is tissue and host specific and is affected by factors such as age and disease^26^. Chondroitin sulfate D is found in the adult human brain, while chondroitin sulfate A and C are typically found in cartilage and other tissues^26^.

In this study, for the first time, we present a quantitative measure of the binding affinity to the known ligands of NHBA, heparin and heparan sulfate, and also to the newly discovered glycan structures. The highest affinity interaction was between NHBA and chondroitin sulfate (K_D_ = 5.2 nM). The chondroitin sulfate used is a natural product that contains a mixture of sulfation patterns (chondroitin sulfate A, C, D). Therefore, alternative sources of chondroitin sulfate polymers were also used to address the question of whether the alternate sulfation patterns played a role in NHBA affinity. Results indicate that NHBA prefers chondroitin sulfate D (2S on GlcA/IdoA and GalNAc with 6S), as there was a 10,000-fold decrease in affinity to chondroitin sulfate C and no binding to chondroitin sulfate A or B. A source of pure chondroitin sulfate D was not available, however studies using the repeating subunit disaccharide structures that make up the chondroitin sulfate polymer confirmed that the chondroitin sulfate type D disaccharide was the preferred substrate with a K_D_ of 9.5 nM. This is a remarkably high affinity considering that it is a small disaccharide subunit that does not have the polyvalency of high molecular weight chondroitin sulfate polymer.

The affinity of NHBA for chondroitin sulfate is 10-fold higher than for the previously described ligand heparin (K_D_ = 52 nM), and 200-fold higher than for heparan sulfate (K_D_ = 1.362 µM). It is interesting to consider that the binding to heparin with an affinity of 52 nM can only be achieved with the polymer. This is unlike chondroitin sulfate, which has nanomolar affinity for both polymer and disaccharide. Heparin disaccharides all had affinities approximately 500-fold weaker (K_D_ > 2.5 µM) than we observed for the equivalent chondroitin sulfate disaccharide. Unlike chondroitin sulfate, this indicates the high affinity interaction observed in SPR with heparin requires polyvalent binding. Previous studies have indicated that NHBA-mediated adherence to epithelial cells (endometrial adenocarcinoma cell line called Hec1-B) is heparan sulfate-dependent and chondroitin sulfate-independent^12^. This observation is not consistent with the high affinity NHBA-chondroitin sulfate interaction that we observed herein. However, the expression of different chondroitin sulfate types is variable between different tissues^26^ and all cancer cell lines that have been investigated do not express the preferred NHBA ligand, chondroitin sulfate D^30,31^. As a result, one would not expect to see NHBA binding to the atypical chondroitin sulfate types expressed by transformed cell lines typically used in cell association studies.

NHBA has a positively charged, arginine-rich region that is required for binding to heparin^11^ and heparan sulfate^12^. When the phase variable meningococcal protease NalP is expressed, NHBA is cleaved and the Arg-rich C terminal fragment is released^11^. The C terminal fragment has been shown to alter endothelial permeability and promote vascular leakage^32^, but its target receptor on endothelial cells is unclear. Competition by SPR revealed that the region of NHBA where chondroitin sulfate D disaccharide binds is also bound by heparin. However, this region does not overlap with the heparan sulfate binding region. Interestingly, the binding of heparin and heparan sulfate seem to overlap, suggesting that there may be two binding sites; a specific glycan recognizing pocket that binds to a preferred disaccharide portion of the GAGs, and a positively charged region that increases the affinity of binding to the rest of the polymer.

The only other high affinity NHBA interaction in the nanomolar range that was observed was with a non-GAG structure, the ganglioside GT3 (K_D_ = 210 nM) that is transiently expressed in the brain during embryonic development^33^ and is found on adult oligodendrocyte progenitor cells in the human adult brain^34^. GT3 consists of three negatively charged side branching sialic acid residues that may mimic the presentation of the sulfated GAGs and explain the binding of NHBA to this otherwise unrelated structure.

The interaction between NHBA and DNA is also likely to be charge dependent, since binding does not appear to be linked to a specific DNA sequence, with multiple NHBA proteins binding to a single double stranded piece of DNA. Future structural studies are required to determine the role of specific NHBA regions, including the Arg-rich region, in binding to the newly identified glycan structures and to DNA.

The ability of *N. meningitidis* to bind several glycan structures suggests that NHBA may target different host glycans in different microenvironments. However, the high affinity of NHBA-chondroitin sulfate D interactions suggest NHBA has evolved to preferentially bind this structure, indicating that it may have a key and unique role in meningococcal pathogenesis. If vaccine induced antibodies are able to block NHBA- chondroitin sulfate D interactions, then this may increase the efficacy of the vaccine.

## Materials and Methods

### Bacterial strains and growth conditions

*N. meningitidis* Ȼ3 (a *siaD* knockout mutant derived from the serogroup B clinical isolate MC58^18^) and the isogenic Ȼ3∆*nhbA* mutant (generated as described previously^11^) were grown on BHI supplemented with 10% Levinthal’s base and incubated overnight at 37 °C at 5% CO_2_. Levinthal’s base is prepared by adding defibrinated horse blood to three volumes of BHI broth, heating at 95 °C for 40 mins, followed by centrifugation (4500 × g, 4 °C, 15 min) to remove to remove insoluble material from the lysed blood.

### Expression of recombinant NHBA

The *nhbA* gene was amplified from *N. meningitidis* MC58 (primers 5′-attactcgagTCGCCCGATGTCAAGTC-3′ and 5′-tgaaatgcatCGGCATCAACATCAATC-3′; underline indicates restriction enzyme sites) and cloned into pET19b (Invitrogen). Protein expression and purification was performed as described previously^35^.

### Glycan array analysis

Glycan array analysis was performed as per Mubaiwa *et al*. using array v3.0 from the Institute for Glycomics and a ProScan Array Microarray scanner (Perkin Elmer) 555ex/568em^19^. Briefly, 125 μL of Bodipy558-succinimidyl ester labelled bacteria (OD 600 = 0.1) were added to the array slide and hybridized for 30 min. Analysis of recombinant NHBA was performed as per Day *et al*.^36^, using 1–2 μg of His-tagged protein. Binding was defined as positive if the average fluorescence intensity of the glycan spots was greater than one-fold above the adjusted background (average of the slide background plus three standard deviations) in three independent replicates (Student’s t-test p < 0.001).

### SPR analysis of recombinant protein or DNA

SPR was performed using a BIAcore T200 instrument and Series S CM5 sensor chips (GE Healthcare). Recombinant NHBA (100 µg/ml) was immobilized by amine coupling (10 µL/min flow rate onto flow cells 2–4), resulting in ~8000 response units (RU) of immobilized protein. The reference surface (flow cell 1) underwent the same treatment, without protein injection. Single cycle kinetics was used to generate the affinity (K_D_) of each interaction, with analytes run in a 1:5 dilution series at concentrations ranging between 1 nM to 100 µM. Glycans or DNA were injected at a flow rate of 10 μl/min, or 30 μl/min for polymers larger than 10 kDa, with a contact time of 60 sec and a dissociation time of 600 sec. Duplicate datasets was analyzed using the BIAcore T200 evaluation software 2.0.2; sensorgrams were double reference subtracted.

### SPR NHBA competition assays

SPR competition assays were performed by using a BIAcore S200 instrument and the A-B-A inject function. Recombinant NHBA was immobilized as above. The A-B-A was used with combinations of each of the glycan substrates (at concentration 10 × K_D_) and PBS controls, with 120 second injections of analyte A to ensure saturation or near-saturation was reached prior to competition with analyte B. The results were analyzed using BIAcore S200 evaluation software.

### ITC analysis of NHBA with DNA

A 503 bp DNA fragment was amplified from *N. meningitidis* genomic DNA (using primers 5′-GGTCGTGTTCCAAAAGACGGG-3′ and 5′-GAAGCCTGAATGCCTTTCCAGC-3′), cleaned up using a QIAGEN PCR purification kit and quantified using a nanodrop. ITC was performed using a nano-ITC (TA instruments) with 10 µM NHBA titrated into 1 µM of DNA (20 injections of 2.5 µL NHBA at 300 second intervals). PBS-NHBA interactions were run as a negative control. This was also background subtracted. Affinity data was determined for a minimum of two repeats and the average K_D_ values and stoichiometry (n) are reported.

## Electronic supplementary material

Supplementary information
