# Supplementary material for: The Bexsero Neisseria meningitidis serogroup B vaccine antigen NHBA is a high-affinity chondroitin sulfate binding protein
Source: Sci Rep. 2018 Apr 25;8:6512. doi: 10.1038/s41598-018-24639-x (PMC5916922; doi:10.1038/s41598-018-24639-x)

The Bexsero *Neisseria meningitidis* serogroup B vaccine antigen NHBA is a high-affinity chondroitin sulfate binding protein.

Tsitsi D. Mubaiwa, Lauren E. Hartley-Tassell, Evgeny A. Semchenko, Christopher J. Day, Michael P. Jennings, Kate L. Seib*

Institute for Glycomics, Griffith University, Gold Coast, Queensland, Australia

* Address correspondence to k.seib@griffith.edu.au

Supplementary material includes:

Table S1: Glycan array results of *Neisseria meningitidis* MC58 whole cells and recombinant NHBA (rNHBA) protein.

Dataset S1. Representative sensorgrams from surface plasmon resonance (SPR) analysis of NHBA – glycan interactions. Graph shows response units (y-axis) over time in deciseconds (x- axis).

Dataset S2. (A) Representative sensorgram from surface plasmon resonance (SPR) analysis of NHBA-DNA interactions. Graph shows response units (y-axis) over time in deciseconds (x- axis). (B) Agarose gel electrophoresis of 503bp PCR product used in SPR.

Supplementary Table S1: Glycan array results of *Neisseria meningitidis* MC58 whole cells and recombinant NHBA (rNHBA) protein.

Glycan			Strain		Protein
**Class**	**Index**	**Structure**	**C3**	**C3 Δ*nhbA***	**rNHBA**
Terminal Gal	2	Galα-sp3			
	3	Galβ-sp3	5.99	2.96	
	37	3-O-Su-Galβ-sp3			
	75	Galα1-2Galβ-sp3	3.82	4.66	2.52
	76	Galα1-3Galβ-sp3	3.23		
	77	Galα1-3GalNAcβ-sp3		1.76	4.28
	78	Galα1-3GalNAcα-sp3	5.37	3.08	
	80	Galα1-3GlcNAcβ-sp3	1.88		
	81	Galα1-4GlcNAcβ-sp3	3.69	3.77	
	83	Galα1-6Glcβ-sp4	3.19	2.11	
	84	Galβ1-2Galβ-sp3		2.60	
	85	Galβ1-3GlcNaAcβ-sp3		1.99	
	87	Galβ1-3Galβ-sp3			
	88	Galβ1-3GalNAcβ-sp3	2.48	1.57	
	89	Galβ1-3GalNAcα-sp3	1.72		
	93	Galβ1-4Glcβ-sp4	7.69	2.62	
	94	Galβ1-4Galβ-sp4	2.61		
	97	Galβ1-4GlcNAcβ-sp3			
	100	Galβ1-6Galβ-sp4			
	145	Galβ1-3(6-O-Su)GlcNAcβ-sp3	5.12	4.19	
	146	Galβ1-4(6-O-Su)Glcβ-sp2	5.58		
	147	Galβ1-4(6-O-Su)GlcNAcβ-sp3	2.48	7.07	1.72
	150	3-O-Su-Galβ1-3GalNAcα-sp3	5.49	4.51	
	151	6-O-Su-Galβ1-3GalNAcα-sp3	6.45	4.87	
	152	3-O-Su-Galβ1-4Glcβ-sp2	3.49	3.91	2.07
	153	6-O-Su-Galβ1-4Glcβ-sp2	4.64		
	155	3-O-Su-Galβ1-3GlcNAcβ-sp3			
	157	3-O-Su-Galβ1-4GlcNAcβ-sp3		3.54	
	159	4-O-Su-Galβ1-4GlcNAcβ-sp3	3.92	4.78	
	161	6-O-Su-Galβ1-3GlcNAcβ-sp3	5.67	3.04	
	163	6-O-Su-Galβ1-4GlcNAcβ-sp3	2.50		
	176	3-O-Su-Galβ1-4(6-O-Su)Glcβ-sp2	4.85	2.35	
	177	3-O-Su-Galβ1-4(6-O-Su)GlcNAcβ-sp2	3.75	4.02	
	178	6-O-Su-Galβ1-4(6-O-Su)Glcβ-sp2	2.46	2.80	
	179	6-O-Su-Galβ1-3(6-O-Su)GlcNAcβ-sp2	4.50	2.90	
	180	6-O-Su-Galβ1-4(6-O-Su)GlcNAcβ-sp2	4.87		
	181	3,4-O-Su$_2$-Galβ1-4GlcNAcβ-sp3	5.39	3.22	
	182	3,6-O-Su$_2$-Galβ1-4GlcNAcβ-sp2	3.53	3.36	
	183	4,6-O-Su$_2$-Galβ1-4GlcNAcβ-sp2	2.56		
	184	4,6-O-Su$_2$-Galβ1-4GlcNAcβ-sp3	3.23	3.13	
	189	3,6-O-Su$_2$-Galβ1-4(6-O-Su)GlcNAcβ-sp2	6.90	4.80	
	201	3,4-O-Su$_2$-Galβ1-4GlcNAcβ-sp3	5.48	2.96	
	203	Galβ1-4(6-O-Su)GlcNAcβ-sp2	5.51	2.16	

220	Galα1-3Galβ1-4Glcβ-sp2	3.83	6.58
222	Galα1-3Galβ1-4GlcNAcβ-sp3	3.83	
224	Galα1-4Galβ1-4Glcβ-sp3		
225	Galα1-4Galβ1-4GlcNAc-sp2		
228	Galβ1-2Galα1-4GlcNAcβ-sp4		2.82
229	Galβ1-3Galβ1-4GlcNAcβ-sp4	3.08	3.50
231	Galβ1-4GlcNAcβ1-3GalNAcα-sp3		2.92
232	Galβ1-4GlcNAcβ1-6GalNAcα-sp3		
254	Galβ1-3(GlcNAcβ1-6)GalNAcα-sp3		2.66
262	Galβ1-3GalNAcβ1-3Gal-sp4	4.74	6.93
264	Galβ1-4Galβ1-4GlcNAc-sp3		3.40
373	Galα1-3Galβ1-4GlcNAcβ1-3Galβ-sp3		4.16
375	Galα1-4GlcNAcβ1-3Galβ1-4GlcNAcβ-sp3	3.51	3.38
376	Galβ1-3GlcNAcβ1-3Galβ1-4Glcβ-sp4		
377	Galβ1-3GlcNAcβ1-3Galβ1-3GlcNAcβ-sp2	3.29	5.83
378	Galβ1-3GlcNAcα1-3Galβ1-4GlcNAcβ-sp3	3.17	2.23
379	Galβ1-3GlcNAcβ1-3Galβ1-4GlcNAcβ-sp3	3.55	4.33
380	Galβ1-3GlcNAcα1-6Galβ1-4GlcNAcβ-sp2	3.31	8.05
381	Galβ1-3GlcNAcβ1-6Galβ1-4GlcNAcβ-sp2		
382	Galβ1-3GalNAcβ1-4Galβ1-4Glcβ-sp3	2.87	1.71
383	Galβ1-4GlcNAcβ1-3Galβ1-4Glcβ-sp2	2.62	4.31
385	Galβ1-4GlcNAcβ1-3Galβ1-4GlcNAcβ-sp3	3.36	3.12
387	Galβ1-4GlcNAcβ1-6Galβ1-4GlcNAcβ-sp2		5.02
388	Galβ1-3(Galβ1-4GlcNAcβ1-6)GalNAcα-sp3	2.67	2.67
401	Galβ1-3GlcNAcβ1-3Galβ1-3GlcNAcβ-sp3		1.83
419	3-O-Su-Galβ1-4GlcNAcβ1-3Galβ1-4GlcNAcβ-sp3	1.99	
420	4-O-Su-Galβ1-4GlcNAcβ1-3Galβ1-4GlcNAcβ-sp3	3.00	4.41
481	Galα1-3Galβ1-4GlcNAcβ1-3Galβ1-4Glcβ-sp4	3.70	8.10
488	Galβ1-4GlcNAcβ1-3(Galβ1-4GlcNAcβ1-6)GalNAcα-sp3	2.12	2.97
489	Galβ1-4GlcNAcβ1-3(GlcNAcβ1-6)Galβ1-4GlcNAc-sp2	2.68	3.06
490	Galβ1-4GlcNAcβ1-6(GlcNAcβ1-3)Galβ1-4GlcNAcβ-sp2	3.66	8.82
498	(Galβ1-4GlcNAcβ1-3)$_3$-sp3	3.26	4.29
499	Galβ1-4GlcNAcβ1-3(Galβ1-4GlcNAcβ1-6)Galβ1-4GlcNAc-sp2		
501	Galβ1-3GalNAcβ1-3Galα1-4Galβ1-4Glcβ-sp4	2.31	3.42
504	(Galβ1-4GlcNAcβ1-4Manα1-3) Galβ1-4GlcNAcβ1-4Manα1-6Manβ1-4GlcNAcβ1-4GlcNAcβ-sp4	2.53	7.50
1A	Galβ1-3GlcNAc	2.89	3.71
1B	Galβ1-4GlcNAc	3.83	1.61
1C	Galβ1-4Gal		
1D	Galβ1-6GlcNAc	6.41	2.50
1E	Galβ1-3GalNAc	4.77	1.89
1F	Galβ1-3GalNAcβ1-4Galβ1-4Glc		
1G	Galβ1-3GlcNAcβ1-3Galβ1-4Glc	5.71	1.78
1H	Galβ1-4GlcNAcβ1-3Galβ1-4Glc		4.27
1I	Galβ1-4GlcNAcβ1-6(Galβ1-4GlcNAcβ1-3)Galβ1-4Glc	4.57	2.38
1J	Galβ1-4GlcNAcβ1-6(Galβ1-3GlcNAcβ1-3)Galβ1-4Glc	6.80	2.34
1K	Galα1-4Galβ1-4Glc		
1L	GalNAcα1-O-Ser		
1M	Galβ1-3GalNAcα1-O-Ser		
1N	Galα1-3Gal	3.79	4.59
1O	Galα1-3Galβ1-4GlcNAc		
1P	Galα1-3Galβ1-4Glc	3.16	1.98

	2A	Galα1-3Galβ1-4Galα1-3Gal	5.44	3.45	
	2B	Galβ1-6Gal	4.72	2.64	
	2C	GalNAcβ1-3Gal	4.54	3.51	3.35
	2D	GalNAcβ1-4Gal	5.65	2.54	
	2E	Galα1-4Galβ1-4GlcNAc	6.19		
	2F	GalNAcα1-3Galβ1-4Glc	4.38		
	2G	Galβ1-3GlcNAcβ1-3Galβ1-4GlcNAcβ1-6(Galβ1-3GlcNAcβ1-3)Galβ1-4Glc	6.17	1.94	3.73
**Terminal GlcNAc**	10	GlcNAcβ-sp3		2.19	
	22	GlcNAcβ-sp4	5.60	3.41	1.99
	43	6-O-Su-GlcNAcβ-sp3	4.10	2.19	
	55	3-O-Su-GlcNAcβ-sp3	4.24		
	113	GlcNAcβ1-3GalNAcα-sp3	3.14	2.60	
	114	GlcNAcβ1-3Manβ-sp4			
	115	GlcNAcβ1-4GlcNAcβ-Asn	2.66	3.07	
	117	GlcNAcβ1-4GlcNAcβ-sp4			
	118	GlcNAcβ1-6GalNAcα-sp3			
	149	GlcNAcβ1-4(6-O-Su)GlcNAcβ-sp2	6.55	3.21	
	167	GlcNAcβ1-4-[HOOC(CH_3)CH]-3-O-GlcNAcβ-sp4	3.53		
	168	GlcNAcβ1--[HOOC(CH_3)CH]-3-O-GlcNAcβ-L-alanyl-D-i-glutaminyl-L-lysine	3.22		
	246	GlcNAcβ1-2Galβ1-3GalNAcα-sp3	3.31	2.84	
	247	GlcNAcβ1-3Galβ1-3GalNAcα-sp3		1.84	
	248	GlcNAcβ1-3Galβ1-4Glcβ-sp2		2.94	
	250	GlcNAcβ1-3Galβ1-4GlcNAcβ-sp3	2.60	2.32	
	251	GlcNAcβ1-4Galβ1-4GlcNAcβ-sp2	3.25	2.48	
	252	GlcNAcβ1-4GlcNAcβ1-4GlcNAcβ-sp4	2.66		
	253	GlcNAcβ1-6Galβ1-4GlcNAcβ-sp2	3.21	3.99	
	255	GlcNAcβ1-3(GlcNAcβ1-6)GalNAcα-sp3	3.45	4.35	
	395	GlcNAcβ1-3(GlcNAcβ1-6)Galβ1-4GlcNAcβ-sp3	2.71	5.38	
	493	(GlcNAcβ1-4)$_5$β-sp4	2.79	2.32	
	503	(GlcNAcβ1-4)$_6$β-sp4	3.03	2.62	
	505	(GlcNAcβ1-4Manα1-3) GlcNAcβ1-4Manα1-6Manβ1-4GlcNAcβ1-4GlcNAcβ-sp4			
	4A	GlcNAcβ1-4GlcNAc	5.41	2.17	
	4B	GlcNAcβ1-4GlcNAcβ1-4GlcNAc	6.17	3.07	
	4C	GlcNAcβ1-4GlcNAcβ1-4GlcNAcβ1-4GlcNAc	4.80	2.86	
	4D	GlcNAcβ1-4GlcNAcβ1-4GlcNAcβ1-4GlcNAcβ1-4GlcNAcβ1-4GlcNAc			
	4E	GlcNAcβ1-4MurNAc	4.78	2.45	3.97
**Mannosyl**	16	Manα-sp3			
	18	Manβ-sp4	3.24		
	19	ManNAcβ-sp4	4.19	3.82	2.00
	47	6-H_2PO_3Manα-sp3	2.46	3.33	1.26
	119	Manα1-2Manβ-sp4	3.03		
	120	Manα1-3Manβ-sp4	4.84		
	121	Manα1-4Manβ-sp4	4.05		
	122	Manα1-6Manβ-sp4	4.71	2.16	
	123	Manβ1-4GlcNAcβ-sp4	4.53		
	124	Manα1-2Manα-sp4	4.42	3.04	
	258	Manα1-3(Manα1-6)Manβ-sp4		2.49	
	495	Manα1-6(Manα1-3)Manα1-6(Manα1-3)Manβ-sp4	3.17	3.41	

	5A	GlcNAcβ1-2Man	3.62	3.34	
	5B	GlcNAcβ1-2Manα1-6(GlcNAcβ1-2Manα1-3)Man	4.75		
	5C	Manα1-2Man			
	5D	Manα1-3Man	6.24		2.39
	5E	Manα1-4Man	7.03	2.71	
	5F	Manα1-6Man	4.44	4.38	
	5G	Manα1-6(Manα1-3)Man		2.28	
	5H	Manα1-6(Manα1-3)Manα1-6(Manα1-3)Man	4.68		
**Fucosyl**	1	Fucα-sp3	3.99	2.09	
	71	Fucα1-2Galβ-sp3	3.21		
	72	Fucα1-3GlcNAcβ-sp3	2.30		
	73	Fucα1-4GlcNAcβ-sp3	4.94	7.66	
	215	Fucα1-2Galβ1-3GlcNAcβ-sp3			
	216	Fucα1-2Galβ1-4GlcNAcβ-sp3			
	217	Fucα1-2Galβ1-3GalNAcα-sp3			
	219	Fucα1-2Galβ1-4Glcβ-sp4	2.91	2.97	
	226	Fucα1-2(Galα1-3)Galβ-sp3	2.81	3.81	
	233	Galβ1-3(Fucα1-4)GlcNAcβ-sp3		3.78	
	234	Fucα1-3(Galβ1-4)GlcNAcβ-sp3			
	235	Fucα1-2(GalNAcα1-3)Galβ-sp3	2.06	2.38	
	287	3-O-Su-Galβ1-3(Fucα1-4)GlcNAcβ-sp3	3.80	3.78	
	288	Fucα1-3(3-O-Su-Galβ1-4)GlcNAcβ-sp3	2.10	1.96	
	359	Fucα1-2(Galα1-3)Galβ1-3GlcNAcβ-sp3			
	360	Fucα1-2(Galα1-3)Galβ1-4GlcNAcβ-sp3	3.66	2.47	
	362	Fucα1-2(Galα1-3)Galβ1-3GalNAcα-sp3	2.00	2.21	
	363	Fucα1-2(Galα1-3)Galβ1-3GalNAcβ-sp3			
	364	Fucα1-3(Galα1-3Galβ1-4)GlcNAcβ-sp3	2.89	2.45	
	366	Fucα1-2(GalNAcα1-3)Galβ1-3GlcNAcβ-sp3			
	368	Fucα1-2(GalNAcα1-3)Galβ1-4GlcNAcβ-sp3	3.45	3.37	
	371	Fucα1-2Galβ1-3(Fucα1-4)GlcNAcβ-sp3			
	372	Fucα1-3(Fucα1-2Galβ1-4)GlcNAcβ-sp3		5.82	
	392	Fucα1-2(GalNAcα1-6)GalNAcα1-6-sp3	1.95		
	479	Fucα1-2Galβ1-3GlcNAcβ1-3Galβ1-4Glcβ-sp4	3.78	7.98	
	480	Fucα1-2Galβ1-3GlcNAcβ1-3Galβ1-4GlcNAcβ-sp2	2.85		
	483	Galα1-3(Fucα1-2)Galβ1-4 (Fucα1-3)GlcNAcβ-sp3	2.32		
	496	Fucα1-2Galβ1-3(Fucα1-4)GlcNAcβ1-3Galβ1-4Glcβ-sp4			
	497	Fucα1-3(Fucα1-2Galβ1-4)GlcNAcβ1-3Galβ1-4Glcβ-sp4		6.21	
	538	Galβ1-4(Fucα1-3)GlcNAcβ1-3(Galβ1-3GlcNAcβ1-6)Galβ1-4Glcβ-sp4	1.80	3.36	
	539	Galβ1-4GlcNAcβ1-6(Fucα1-2Galβ1-3GlcNAcβ1-3)Galβ1-4Glcsp4			
	540	Galβ1-4(Fucα1-3)GlcNAcβ1-6(Neu5Acα2-6Galβ1-3GlcNAcβ1-3)Galβ1-4Glcβ-sp4	3.50	6.59	
	541	Galβ1-4(Fucα1-3)GlcNAcβ1-6(Fucα1-2Galβ1-3GlcNAcβ1-3)Galβ1-4Gal-sp4			
	542	Galβ1-3GlcNAcβ1-3Galβ1-4(Fucα1-3)GlcNAcβ1-6(Galβ1-3GlcNAcβ1-3)Galβ1-4Gal-sp4			
	543	Galβ1-4(Fucα1-3)GlcNAcβ1-6(Fucα1-2Galβ1-3(Fucα1-4)GlcNAcβ1-3)Galβ1-4Gal-sp4	4.76	9.62	
	7A	Fucα1-2Galβ1-3GlcNAcβ1-3Galβ1-4Glc	3.37		
	7B	Galβ1-3(Fucα1-4)GlcNAcβ1-3Galβ1-4Glc	7.12		
	7C	Galβ1-4(Fucα1-3)GlcNAcβ1-3Galβ1-4Glc	4.55	2.37	

	7D	Fucα1-2Galβ1-3(Fucα1-4)GlcNAcβ1-3Galβ1-4Glc	6.80	1.94	
	7E	Galβ1-3(Fucα1-4)GlcNAcβ1-3Galβ1-4(Fucα1-3)Glc	4.31	3.23	
	7F	Fucα1-2Gal	5.15	2.57	2.09
	7G	Fucα1-2Galβ1-4Glc	5.40	2.88	
	7H	Galβ1-4(Fucα1-3)Glc	5.71		
	7I	Galβ1-4(Fucα1-3)GlcNAc	4.80	3.72	
	7J	Galβ1-3(Fucα1-4)GlcNAc	7.51		
	7K	GalNAcα1-3(Fucα1-2)Gal	5.66	3.35	
	7L	Fucα1-2Galβ1-4(Fucα1-3)Glc	2.90	3.71	
	7M	Galβ1-3(Fucα1-2)Gal	6.16	2.77	
	7N	Fucα1-2Galβ1-4(Fucα1-3)GlcNAc	6.44	2.57	
	7O	Fucα1-2Galβ1-3GlcNAc	4.78	2.20	
	7P	Fucα1-2Galβ1-3(Fucα1-4)GlcNAc	4.41	3.99	
	8A	SO_3-3Galβ1-3(Fucα1-4)GlcNAc	5.90		
	8B	SO_3-3Galβ1-4(Fucα1-3)GlcNAc	3.44	4.87	
	8C	Galβ1-3GlcNAcβ1-3Galβ1-4(Fucα1-3)GlcNAcβ1-3Galβ1-4Glc			
	8D	Galβ1-4(Fucα1-3)GlcNAcβ1-6(Galβ1-3GlcNAcβ1-3)Galβ1-4Glc			
	8E	Galβ1-4(Fucα1-3)GlcNAcβ1-6(Fucα1-2Galβ1-3GlcNAcβ1-3)Galβ1-4Glc	4.67	3.52	
	8F	Galβ1-4(Fucα1-3)GlcNAcβ1-6(Fucα1-2Galβ1-3(Fucα1-4)GlcNAcβ1-3)Galβ1-4Glc			
	8G	Galβ1-4GlcNAcβ1-3Galβ1-4(Fucα1-3)Glc			
	8H	Fucα1-2Galβ1-4(Fucα1-3)GlcNAcβ1-3Galβ1-4Glc			
	8I	Fucα1-3Galβ1-4GlcNAcβ1-3Galβ1-4(Fucα1-3)Glc			
	8J	Fucα1-2Galβ1-4(Fucα1-3)GlcNAcβ1-3(Fucα1-2)Galβ1-4Glc			
	8K	Galβ1-4(Fucα1-3)GlcNAcβ1-6(Galβ1-4GlcNAcβ1-3)Galβ1-4Glc			
	8L	Galβ1-4(Fucα1-3)GlcNAcβ1-6(Galβ1-4(Fucα1-3)GlcNAcβ1-3)Galβ1-4Glc			
	8M	Fucα1-2Galβ1-4(Fucα1-3)GlcNAcβ1-6(Galβ1-4GlcNAcβb1-3)Galβ1-4Glc			
	8N	Galβ1-3GlcNAcβ1-3Galβ1-4(Fucα1-3)GlcNAcβ1-6(Galβ1-3GlcNAcβ1-3)Galβ1-4Glc			
	8O	Fucα1-2Galβ1-3GlcNAcβ1-3Galβb1-4(Fucα1-3)GlcNAcβ1-6(Galβ1-3GlcNAcβ1-3)Galβ1-4Glc	3.92		
**Sialylated**	48	Neu5Acα-sp3		1.75	
	49	Neu5Acα-sp9			
	52	Neu5Gcα-sp3	5.66	2.48	
	54	9-NAc-Neu5Acα-sp3	6.66	2.30	
	169	Neu5Acα2-3Galβ-sp3	4.69	2.80	
	170	Neu5Acα2-6Galβ-sp3			3.01
	171	Neu5Acα2-3GalNAcα-sp3			
	172	Neu5Acα2-6GalNAcα-sp3	3.70	2.84	
	174	Neu5Gcα2-6GalNAcα-sp3	2.13		
	186	Neu5Acα2-8Neu5Acα2-sp3	4.08		
	205	Neu5Acα2-6GalNAcβ-sp3			
	206	Neu5Gcα2-3Gal-sp3		2.20	
	289	Galα1-3(Neu5Acα2-6)GalNAcα-sp3	3.89	5.48	
	290	Galβ1-3(Neu5Acα2-6)GalNAcα-sp3			
	292	Neu5Acα2-3Galβ1-3GalNAcα-sp3		1.81	
	293	Neu5Acα2-3Galβ1-4Glcβ-sp3	1.83	3.31	
	294	Neu5Acα2-3Galβ1-4Glcβ-sp4		1.70	
	295	Neu5Acα2-6Galβ1-4Glcβ-sp2	3.37	5.66	

	298	Neu5Acα2-3Galβ1-4GlcNAcβ-sp3	4.85	4.96	
	299	Neu5Acα2-3Galβ1-3GlcNAcβ-sp3	2.84	2.93	
	300	Neu5Acα2-6Galβ1-4GlcNAcβ-sp3		1.95	
	303	Neu5Gcα2-3Galβ1-4GlcNAcβ-sp3	2.94	2.65	
	304	Neu5Gcα2-6Galβ1-4GlcNAcβ-sp3		2.85	
	306	9-NAc-Neu5Acα2-6Galβ1-4GlcNAcβ-sp3		4.66	
	315	Neu5Acα2-3Galβ1-4-(6-O-Su)GlcNAcβ-sp3	3.10	2.11	
	317	Neu5Acα2-3Galβ1-3-(6-O-Su)GalNAcβ-sp3		6.07	
	318	Neu5Acα2-6Galβ1-4-(6-O-Su)GlcNAcβ-sp3	2.45	1.89	
	319	Neu5Acα2-3-(6-O-Su)Galβ1-4GlcNAcβ-sp3	3.09	4.43	
	321	(Neu5Acα2-8)$_3$-sp3		3.02	
	323	Neu5Acα2-6Galβ1-3GlcNAc-sp3			
	324	Neu5Acα2-6Galβ1-3(6-O-Su)GlcNAc-sp3			
	331	Neu5Gcα2-3Galβ1-3GlcNAcβ-sp3		2.79	
	421	Neu5Acα2-3(GalNAcβ1-4)Galβ1-4Glcβ-sp2			
	422	Neu5Acα2-3Galβ1-4GlcNAcβ1-3Galβ-sp3			
	423	Fucα1-3(Neu5Acα2-3Galβ1-4)GlcNAcβ-sp3			
	426	Neu5Acα2-3Galβ1-3(Fucα1-4)GlcNAcβ-sp3	2.54	2.58	
	428	Fucα1-3(Neu5Acα2-3Galβ1-4)6-O-Su-GlcNAcβ-sp3			
	429	Fucα1-3(Neu5Acα2-3(6-O-Su)Galβ1-4)GlcNAcβ-sp3	2.48	4.56	
	433	Neu5Acα2-3Galβ1-3(Neu5Acα2-6)GalNAcα-sp3	3.18	3.03	
	434	Neu5Acα2-8Neu5Acα2-3Galβ1-4Glcβ-sp4	1.95	3.44	
	527	Neu5Acα2-3Galβ1-4GlcNAcβ1-3Galβ1-4GlcNAcβ-sp2	2.72		
	528	Fucα1-3(Neu5Acα2-3Galβ1-4)GlcNAcβ1-3Galβ-sp3	3.89	7.05	
	529	Galβ1-3(Neu5Acα2-6)GlcNAcβ1-3Galβ1-4Glcβ-sp4	3.02	1.94	
	531	Neu5Acα2-8Neu5Acα2-3(GalNAcβ1-4)Galβ1-4Glc-sp2	3.53	3.09	
	532	Neu5Acα2-8Neu5Acα2-8Neu5Acα2-3Galβ1-4Glc-sp2		3.49	2.63
	533	Neu5Acα2-8Neu5Acα2-8 Neu5Acα2-3(GalNAcβ1-4)Galβ1-4Glc-sp2			
	534	Neu5Acα2-3Galβ1-4GlcNAcβ1-3Galβ1-4GlcNAcβ-sp3	3.86	4.16	
	536	Neu5Acα2-3Galβ1-3GlcNAcβ1-3Galβ1-4Glcβ-sp4	1.92		
	537	Neu5Acα2-3Galβ1-4GlcNAcβ1-3Galβ1-4Glcβ-sp4	3.00	3.67	
	540	Galβ1-4(Fucα1-3)GlcNAcβ1-6(Neu5Acα2-6Galβ1-3GlcNAcβ1-3)Galβ1-4Glcβ-sp4-sp4	3.50	6.59	
	627	Neu5Acα2-6Galβ1-4GlcNAcβ1-4Manα1-3(Neu5Acα2-6Galβ1-4GlcNAcβ1-4Manα1-6)Manβ1-4GlcNAcβ1-4GlcNAcβ-sp4			
	10A	Neu5Acα2-3Galβ1-3(Fucα1-4)GlcNAc			
	10B	Neu5Acα2-3Galβ1-4(Fucα1-3)GlcNAc			
	10C	Neu5Acα2-3Galβ1-3GlcNAcβ1-3Galβ1-4Glc			
	10D	Galβ1-4(Fucα1-3)GlcNAcβ1-6(Neu5Acα2-6Galβ1-4GlcNAcβ1-3)Galβ1-4Glc			
	10E	Neu5Acα2-3Galβ1-3(Neu5Acα2-6)GalNAc			
	10H	Neu5Acα2-6Galβ1-3GlcNAcβ1-3Galβ1-4(Fucα1-3)Glc (SLNFPVI)	3.04		
	10K	Neu5Acα2-3Galβ1-4GlcNAc			
	10L	Neu5Acα2-6Galβ1-4GlcNAc	4.08	2.90	
	10M	Neu5Acα2-3Galβ1-3GlcNAcβ1-3Galβ1-4Glc	4.01		
	10N	Galβ1-3(Neu5Acα2-6)GlcNAcβ1-3Galβ1-4Glc		2.08	
	10O	Neu5Acα2-6Galβ1-4GlcNAcβ1-3Galβ1-4Glc			
	10P	Neu5Acα2-3Galβ1-3(Neu5Acα2-6)GlcNAcβ1-3Galβ1-4Glc	4.61		
	11A	Neu5Acα2-3Galβ1-4Glc			
	11B	Neu5Acα2-6Galβ1-4Glc			

	11C	(Neu5Acα2-8Neu5Ac)n (n<50)	4.80	2.96	
	11D	Neu5Acα2-6Galβ1-4GlcNAcβ1-2Manα1-6(Neu5Acα2-6Galβ1-4GlcNAcβ1-2Manα1-6)Manβ1-4GlcNAcβ1-4GlcNAc-Asn			
**Terminal GalNAc**	4	GalNAcα-sp0			
	5	GalNAcα-sp3	4.25		
	6	GalNAcβ-sp3	2.42	2.06	
	38	3-O-Su-GalNAcα-sp3		2.79	
	101	GalNAcα1-3GalNAcβ-sp3			
	102	GalNAcα1-3Galβ-sp3	3.51	2.24	
	103	GalNAcα1-3GalNAcα-sp3	8.12	3.87	
	104	GalNAcβ1-3Galβ-sp3			
	106	GalNAcβ1-4GlcNAcβ-sp3		3.38	
	192	GalNAcβ1-4(6-O-Su)GlcNAcβ-sp3	4.89	3.35	
	193	3-O-Su-GalNAcβ1-4GlcNAcβ-sp3	4.90	4.32	
	194	6-O-Su-GalNAcβ1-4GlcNAcβ-sp3	3.59	3.81	
	195	6-O-Su-GalNAcβ1-4-(3-O-Su)GlcNAcβ-sp3	5.93	2.93	
	196	3-O-Su-GalNAcβ1-4(3-O-Su)-GlcNAcβ-sp3			
	197	3,6-O-Su$_2$-GalNAcβ1-4GlcNAcβ-sp3	3.53		
	198	4,6-O-Su$_2$-GalNAcβ1-4GlcNAcβ-sp3	2.73	2.57	
	199	4,6-O-Su$_2$-GalNAcβ1-4-(3-O-Ac)GlcNAcβ-sp3			
	200	4-O-Su-GalNAcβ1-4GlcNAcβ-sp3	3.89	1.80	
	201	3,4-O-Su$_2$-Galβ1-4GlcNAcβ-sp3	5.48	2.96	
	202	6-O-Su-GalNAcβ1-4(6-O-Su)GlcNAcβ-sp3			
	204	4-O-Su-GalNAcβ1-4GlcNAcβ-sp2			
	238	GalNAcβ1-4Galβ1-4Glcβ-sp3	2.84	3.57	
	389	GalNAcβ1-3Galα1-4Galβ1-4Glcβ-sp3			
**Terminal Glucose**	7	Glcα-sp3	2.68	1.44	
	9	Glcβ-sp3		3.43	
	46	6-H_2PO_3Glcβ-sp4	2.44		1.67
	110	Glcα1-4Glcβ-sp3			
	111	Glcβ1-4Glcβ-sp4	6.29	3.64	
	112	Glcβ1-6Glcβ-sp4			
	240	(Glcα1-4)$_3$β-sp4	3.31	2.51	
	241	(Glcα1-6)$_3$β-sp4			
	390	(Glcα1-4)$_4$β-sp4	2.78	4.92	
	391	(Glcα1-6)$_4$β-sp4	1.87	3.26	
	492	(Glcα1-6)$_5$β-sp4		4.10	
	502	(Glcα1-6)$_6$β-sp4			
**GAG digests**	12A	Neocarratetraose-41, 3-di-*O*-sulphate (Na^+)	3.38		
	12B	Neocarratetraose-41-*O*-sulphate (Na^+)	4.08		3.33
	12C	Neocarrahexaose-24,41, 3, 5-tetra-*O*-sulphate (Na^+)	3.90		
	12D	Neocarrahexaose-41, 3, 5-tri-*O*-sulphate (Na^+)			
	12E	Neocarraoctaose-41, 3, 5, 7-tetra-*O*-sulphate (Na^+)			
	12F	Neocarradecaose-41, 3, 5, 7, 9-penta-*O*-sulphate (Na^+)			
	12G	ΔUA→2S GlcNS-6S Na_4 (I-S)			
	12H	ΔUA → GlucNS-6S Na_3 (II-S)			
	12I	ΔUA → 2S-GlcNS Na_3 (III-S)			
	12J	ΔUA → 2S-GlcNAc-6S Na_3 (I-A)	3.26		
	12K	ΔUA → GlcNAc-6S Na_2 (II-A)	4.02		
	12L	ΔUA → 2S-GlcNAc Na_2 (III-A)			
	12M	ΔUA → GlcNAc Na (IV-A)	1.67		

	12N	ΔUA → GalNAc-4S Na_2 (Δ Di-4S)			
	12O	ΔUA → GalNAc-6S Na_2 (Δ Di-6S)	5.26		
	12P	ΔUA → GalNAc-4S,6S Na_3 (Δ Di-disE)		3.83	
	13A	ΔUA → 2S-GalNAc-4S Na_2 (Δ Di-disB)	3.81		
	13B	ΔUA → 2S-GalNAc-6S Na_3 (Δ Di-disD)	4.07		1.53
	13C	ΔUA →2S-GalNAc-4S-6S Na_4 (Δ Di-tisS)			
	13D	ΔUA → 2S-GalNAc-6S Na_2 (ΔDi-UA2S)			
	13E	ΔUA → GlcNAc Na (Δ Di-HA)	4.17		2.93
**GAGs (HMW)**	13F	(GlcAβ1-3GlcNAcβ1-4)n (n=4)			
	13G	(GlcAβ1-3GlcNAcβ1-4)n (n=8)			
	13H	(GlcAβ1-3GlcNAcβ1-4)n (n=10)			2.80
	13I	(GlcAβ1-3GlcNAcβ1-4)n (n=12)			
	13J	(GlcA/IdoAα/β1-4GlcNAcα1-4)n (n=200)	3.86		1.95
	13K	(GlcA/IdoAβ1-3(±4/6S)GalNAcβ1-4)n (n<250)	4.85	2.75	1.98
	13L	((±2S)GlcA/IdoAα/β1-3(±4S)GalNAcβ1-4)n (n<250)	3.10		
	13M	(GlcA/IdoAβ1-3(±6S)GalNAcβ1-4)n (n<250)			2.69
	13N	(GlcAβ1-3GlcNAcβ1-4)n (n=4)	4.94	3.70	2.83
	13O	(GlcAβ1-3GlcNAcβ1-4)n (n=6)	5.76	3.99	
	13P	(GlcAβ1-3GlcNAcβ1-4)n (n=8)	3.68		
	14A	(GlcAβ1-3GlcNAcβ1-4)n (n=10)			
	14B	(GlcAβ1-3GlcNAcβ1-4)n (n=12)			
	14C	(GlcAβ1-3GlcNAcβ1-4)n (n=14)		2.79	
	14D	(GlcAβ1-3GlcNAcβ1-4)n (n=16)	3.34	2.70	
	14E	HA-30,000da 2.5mg/ml			
	14F	HA-107,000da 2.5mg/ml	5.45		2.67
	14G	HA-190,000da 2.5mg/ml	3.89	3.08	3.52
	14H	HA-220,000da 2.5mg/ml	6.12	3.60	2.47
	14I	HA-1,600,000da 2.5mg/ml	4.92	2.85	
	14J	Heparan Sulfate 5mg/ml			2.24
	14K	(Glcβ1-3Glcβ1-3)n	2.64	2.41	2.79
**Other**	14	GlcN(Gc)β-sp4	5.65	3.32	
	15	$HOCH_2(HOCH)_4CH_2NH_2$			
	20	Rhaα-sp3		1.72	
	44	GlcAα-sp3			
	45	GlcAβ-sp3		1.99	
	164	GlcAβ1-3GlcNAcβ-sp3	4.78	4.54	
	165	GlcAβ1-3Galβ-sp3	4.07		
	166	GlcAβ1-6Galβ-sp3		2.28	
	625	$(GlcA\beta1\text{-}4GlcNAc\beta1\text{-}3)_8\text{-}NH_2\text{-}ol$	4.47	2.99	

Red represents binding (in three independent replicates) and white is no binding observed. Binding was defined as a value greater than 1-fold increase above mean background relative fluorescence units (RFU). Fold-increase above background is indicated. GAG, glycosaminoglycan; HMW, high molecular weight; HA, hyaluronin; ΔUA, unsaturated uronic aid. Spacers used are: sp2, $(CH_2)_3$-NH- ; sp3, $(CH_2)_5$-NH- ; sp4, NH-(CO)CH_2-NH- .

Supplementary dataset S1. Representative sensorgrams from surface plasmon resonance (SPR) analysis of NHBA – glycan interactions. Graphs show response units (y-axis) over time in deciseconds (x- axis).

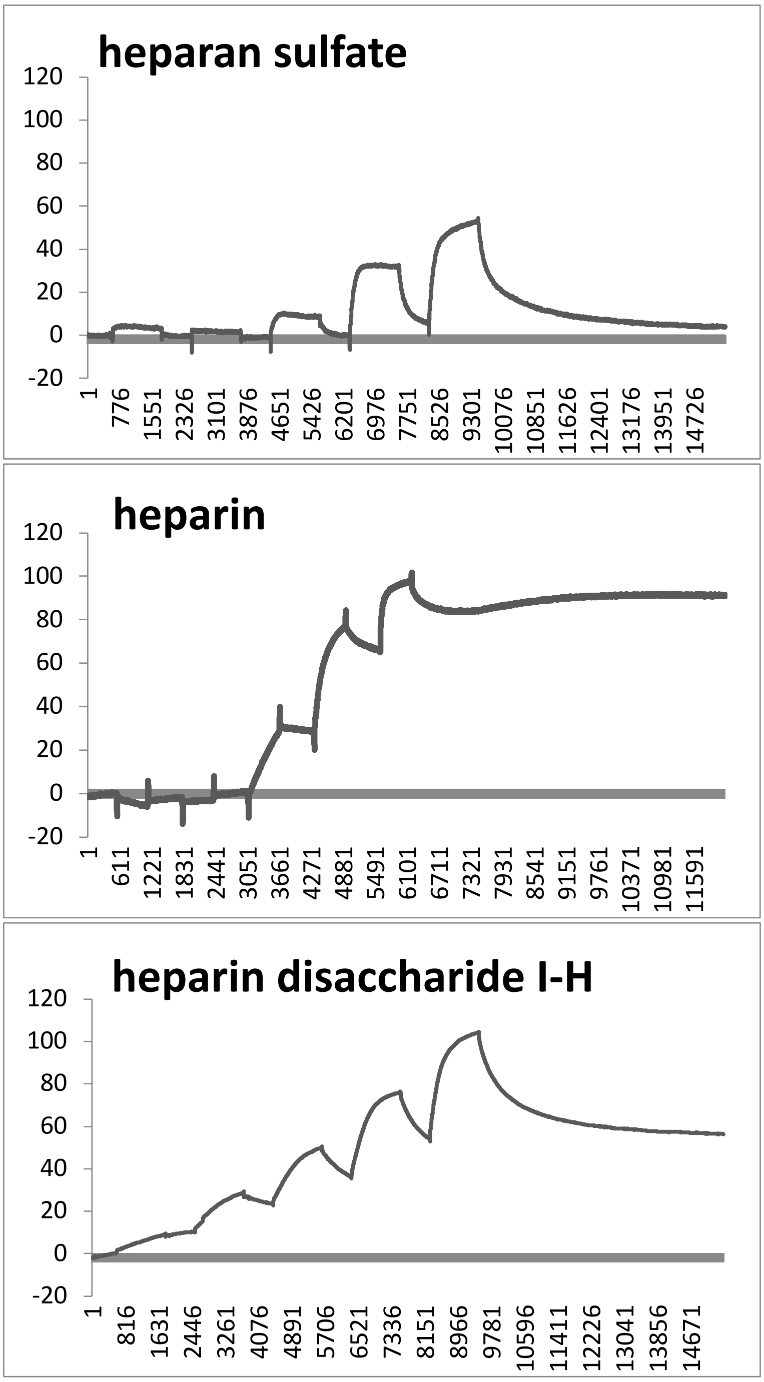

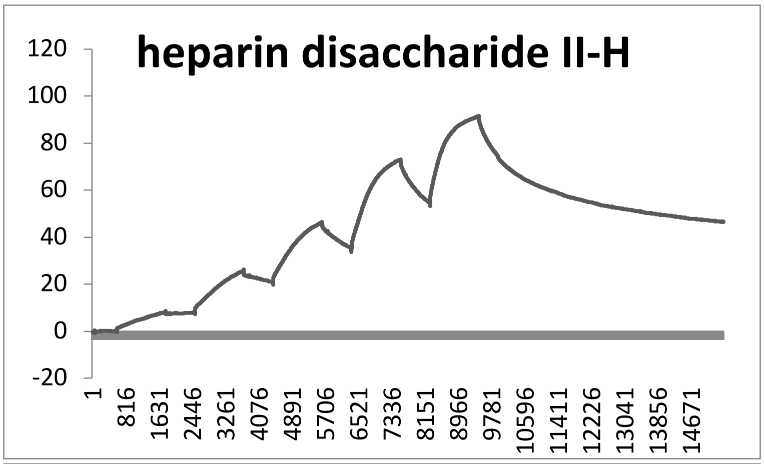
heparin disaccharide II-H
120
100
80
60
40
20
0
-20
1
816
1631
2446
3261
4076
4891
5706
6521
7336
8151
8966
9781
10596
11411
12226
13041
13856
14671

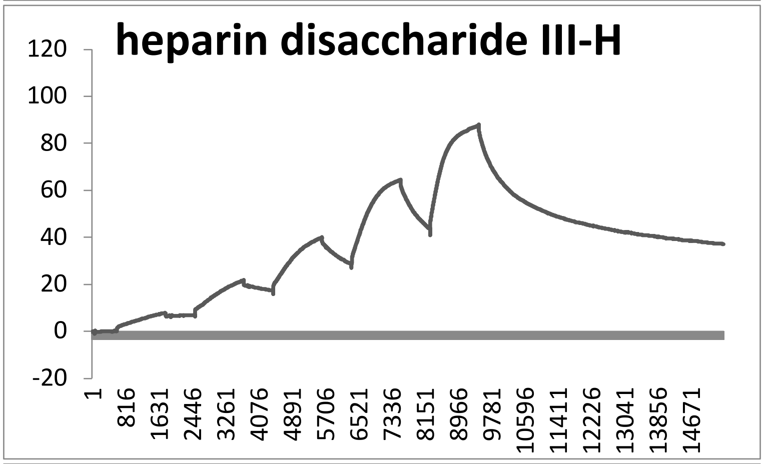
heparin disaccharide III-H
120
100
80
60
40
20
0
-20
1
816
1631
2446
3261
4076
4891
5706
6521
7336
8151
8966
9781
10596
11411
12226
13041
13856
14671

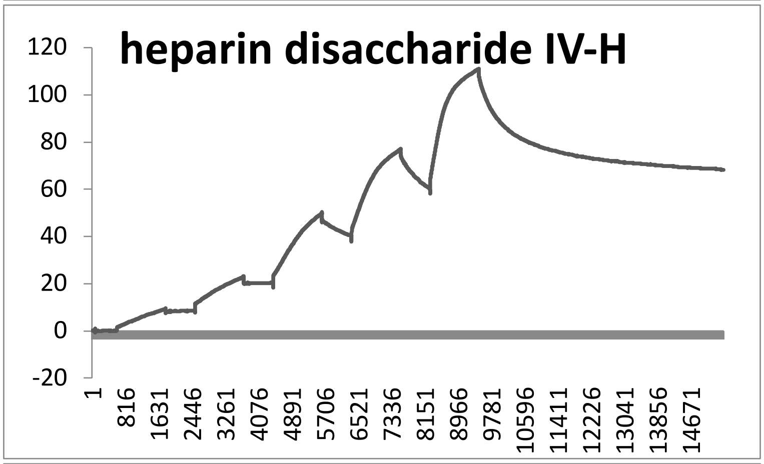
heparin disaccharide IV-H
120
100
80
60
40
20
0
-20
1
816
1631
2446
3261
4076
4891
5706
6521
7336
8151
8966
9781
10596
11411
12226
13041
13856
14671

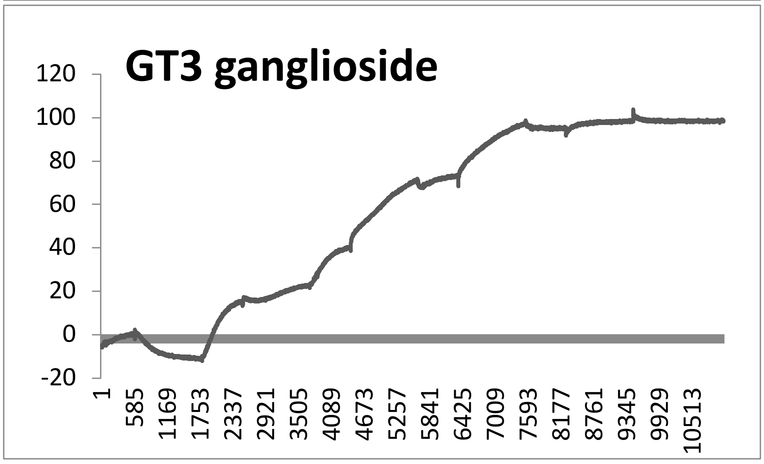
GT3 ganglioside
120
100
80
60
40
20
0
-20
1
585
1169
1753
2337
2921
3505
4089
4673
5257
5841
6425
7009
7593
8177
8761
9345
9929
10513

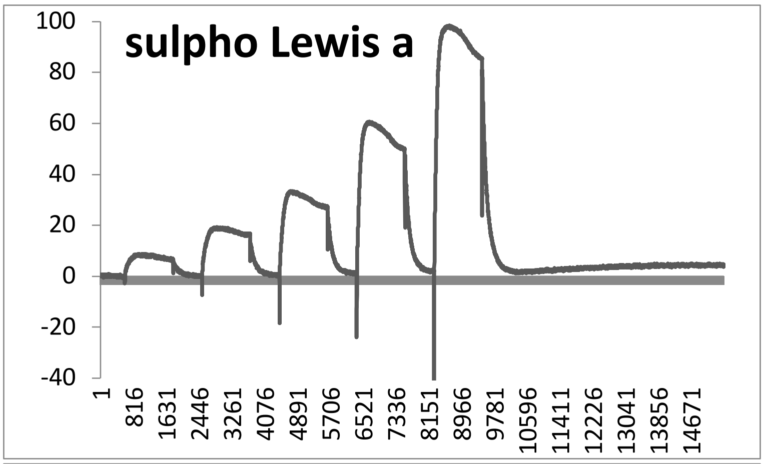
sulpho Lewis a
100
80
60
40
20
0
-20
-40
1
816
1631
2446
3261
4076
4891
5706
6521
7336
8151
8966
9781
10596
11411
12226
13041
13856
14671

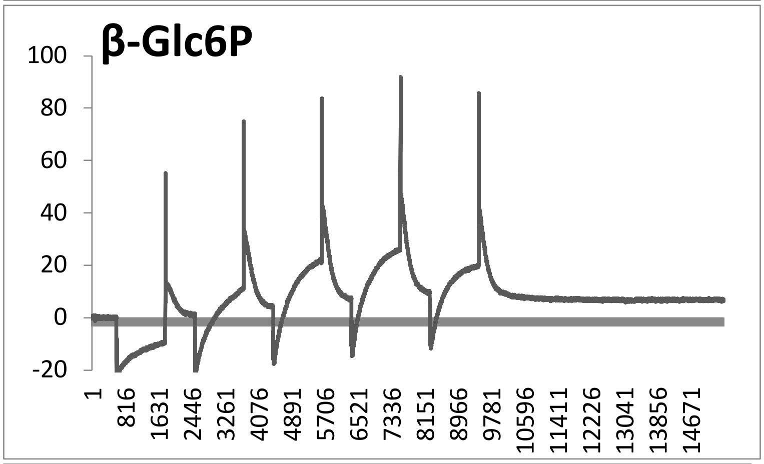
β-Glc6P
100
80
60
40
20
0
-20
1
816
1631
2446
3261
4076
4891
5706
6521
7336
8151
8966
9781
10596
11411
12226
13041
13856
14671

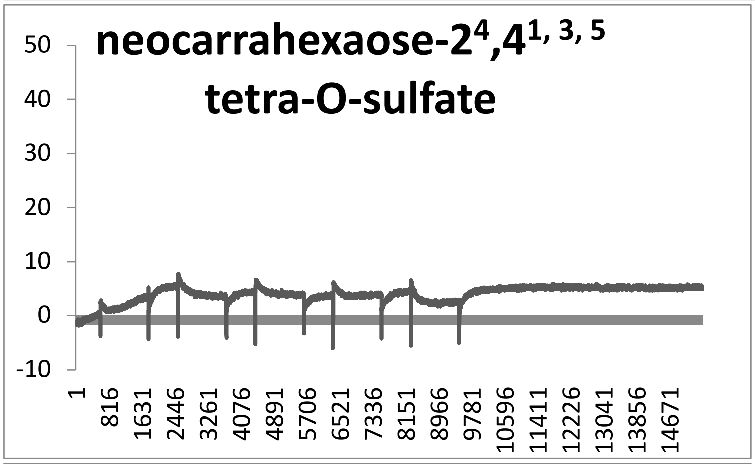
neocarrahexaose-2⁴,4¹,³,⁵ tetra-O-sulfate
50
40
30
20
10
0
-10
1
816
1631
2446
3261
4076
4891
5706
6521
7336
8151
8966
9781
10596
11411
12226
13041
13856
14671

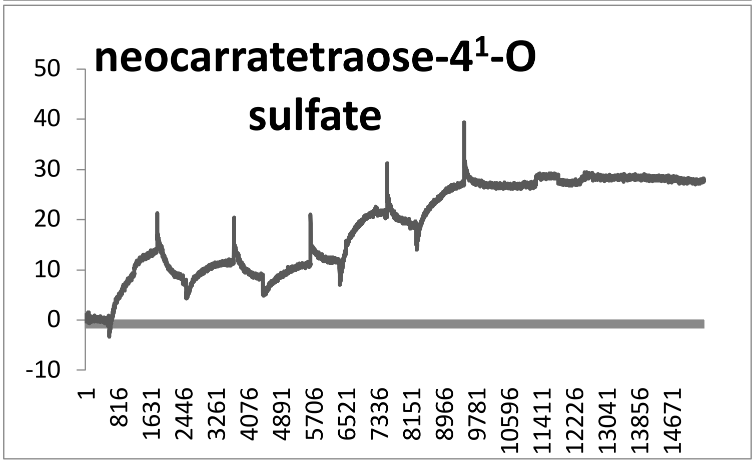
neocarratetraose-4¹-O sulfate
50
40
30
20
10
0
-10
1
816
1631
2446
3261
4076
4891
5706
6521
7336
8151
8966
9781
10596
11411
12226
13041
13856
14671

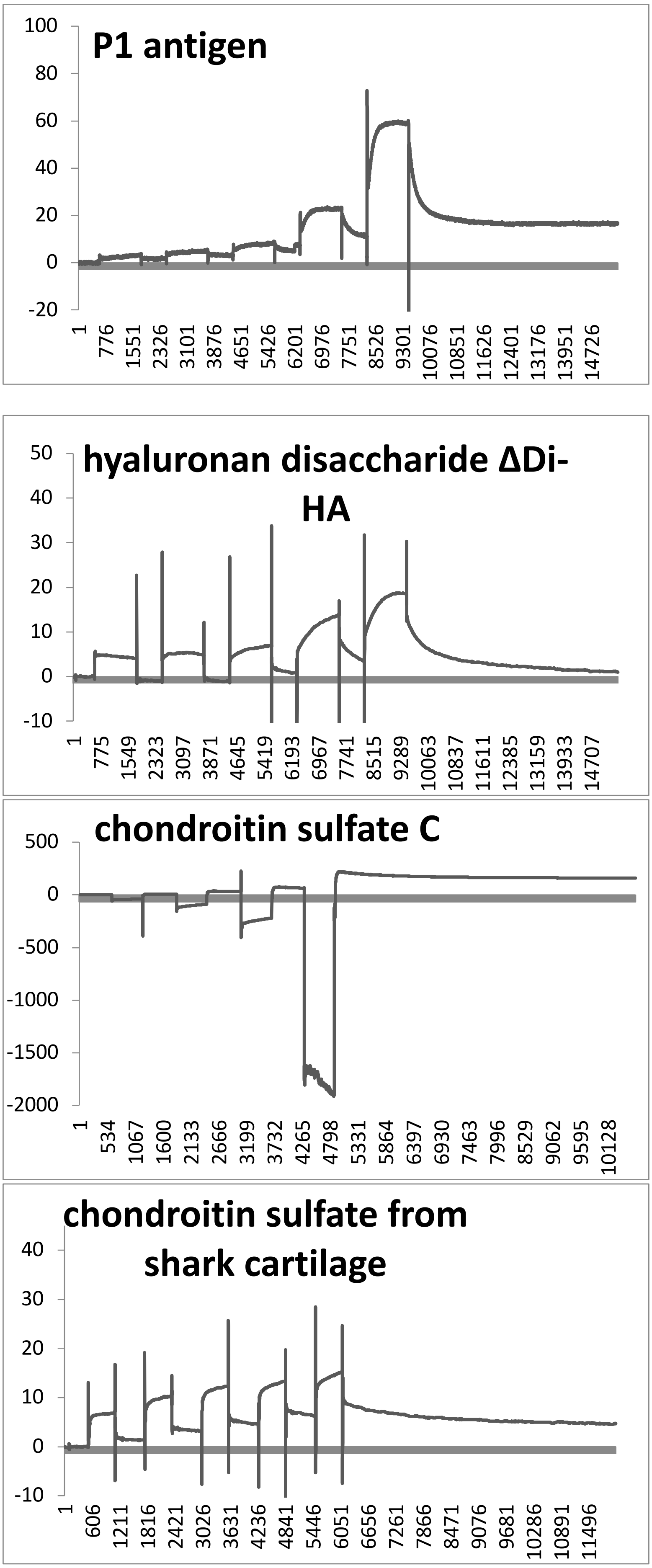

P1 antigen
100
80
60
40
20
0
-20
1
776
1551
2326
3101
3876
4651
5426
6201
6976
7751
8526
9301
10076
10851
11626
12401
13176
13951
14726
hyaluronan disaccharide ΔDi-HA
50
40
30
20
10
0
-10
1
775
1549
2323
3097
3871
4645
5419
6193
6967
7741
8515
9289
10063
10837
11611
12385
13159
13933
14707
chondroitin sulfate C
500
0
-500
-1000
-1500
-2000
1
534
1067
1600
2133
2666
3199
3732
4265
4798
5331
5864
6397
6930
7463
7996
8529
9062
9595
10128
chondroitin sulfate from shark cartilage
40
30
20
10
0
-10
1
606
1211
1816
2421
3026
3631
4236
4841
5446
6051
6656
7261
7866
8471
9076
9681
10286
10891
11496

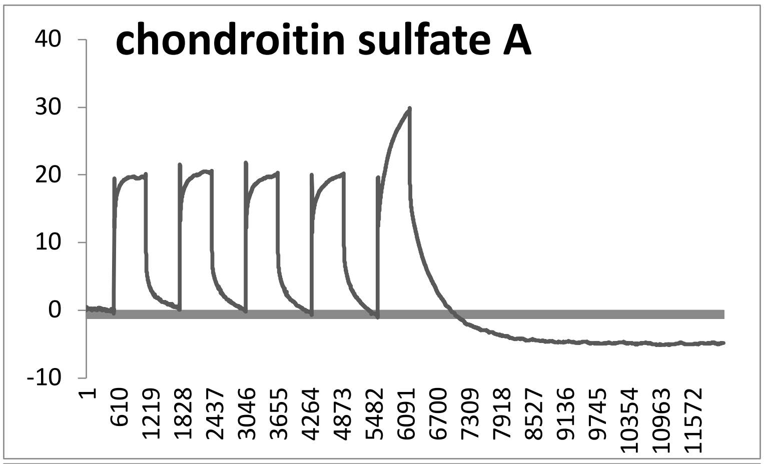
chondroitin sulfate A
40
30
20
10
0
-10
1
610
1219
1828
2437
3046
3655
4264
4873
5482
6091
6700
7309
7918
8527
9136
9745
10354
10963
11572

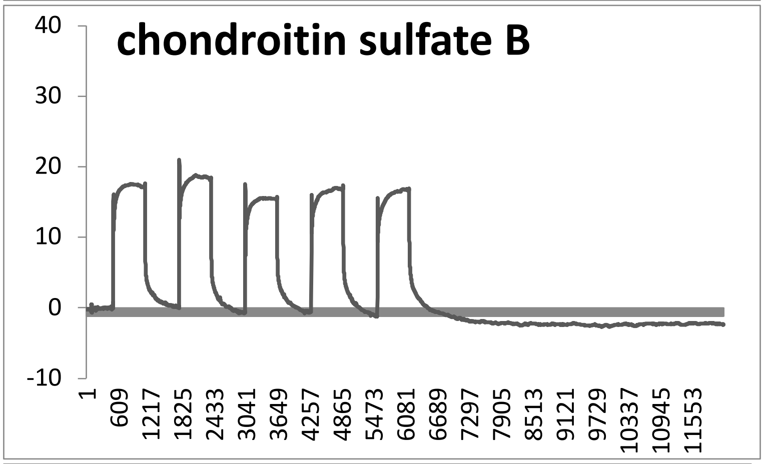
chondroitin sulfate B
40
30
20
10
0
-10
1
609
1217
1825
2433
3041
3649
4257
4865
5473
6081
6689
7297
7905
8513
9121
9729
10337
10945
11553

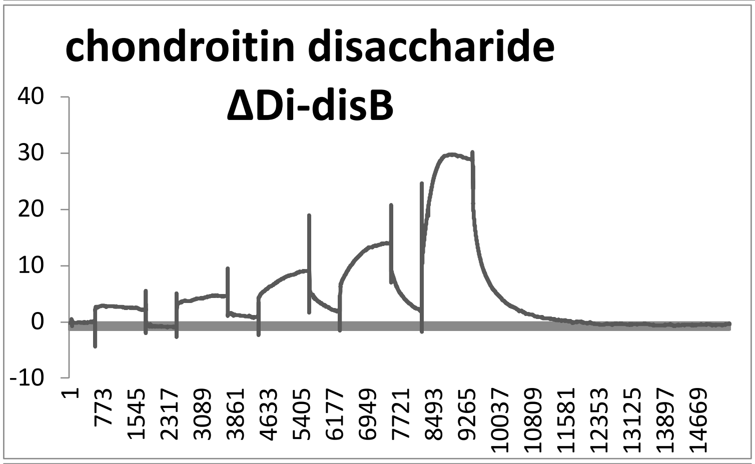
chondroitin disaccharide
ΔDi-disB
40
30
20
10
0
-10
1
773
1545
2317
3089
3861
4633
5405
6177
6949
7721
8493
9265
10037
10809
11581
12353
13125
13897
14669

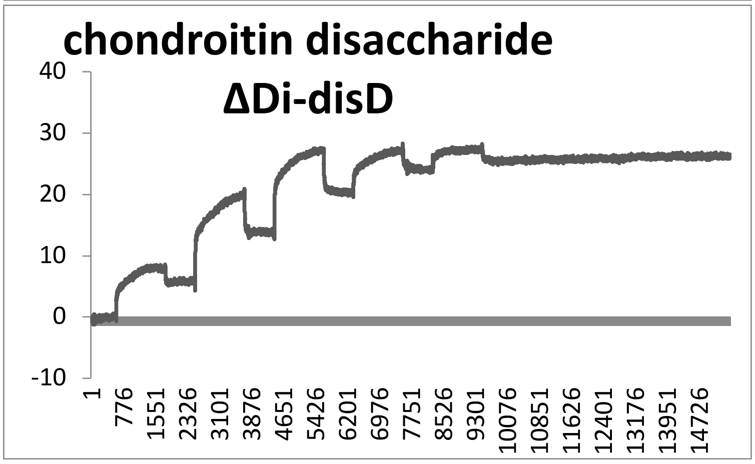
chondroitin disaccharide
ΔDi-disD
40
30
20
10
0
-10
1
776
1551
2326
3101
3876
4651
5426
6201
6976
7751
8526
9301
10076
10851
11626
12401
13176
13951
14726

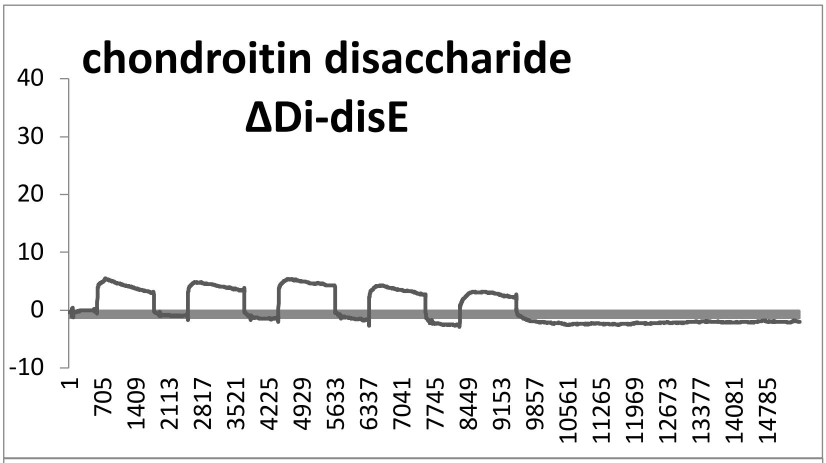
chondroitin disaccharide
ΔDi-disE
40
30
20
10
0
-10
1
705
1409
2113
2817
3521
4225
4929
5633
6337
7041
7745
8449
9153
9857
10561
11265
11969
12673
13377
14081
14785

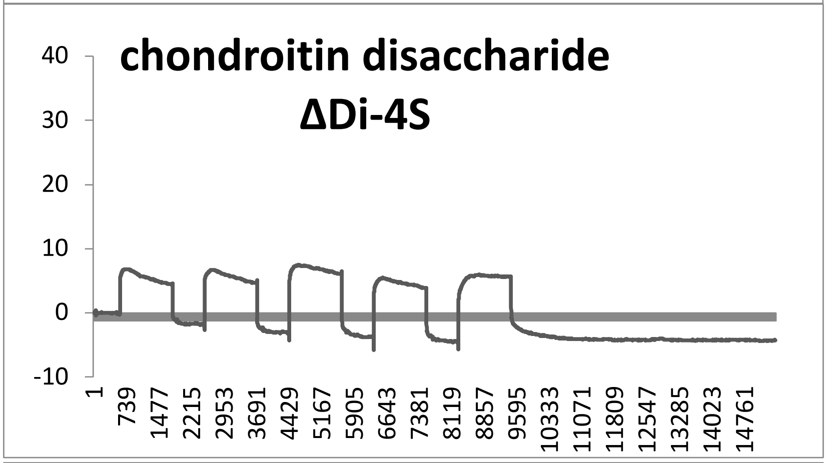
chondroitin disaccharide
ΔDi-4S
40
30
20
10
0
-10
1
739
1477
2215
2953
3691
4429
5167
5905
6643
7381
8119
8857
9595
10333
11071
11809
12547
13285
14023
14761

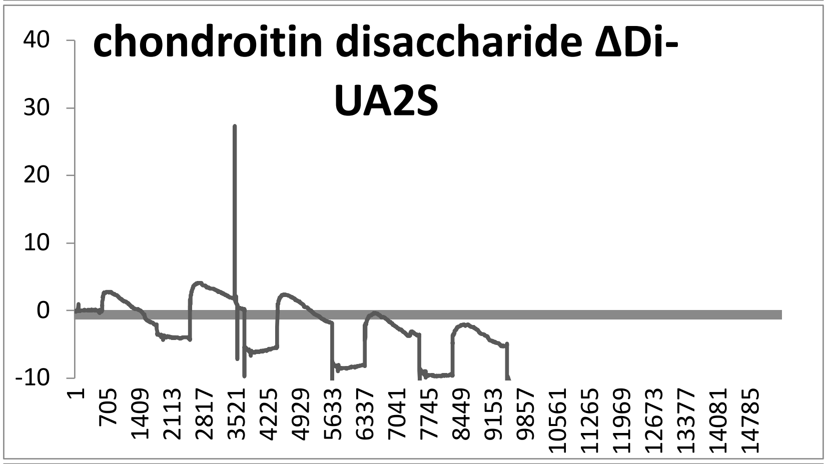
chondroitin disaccharide ΔDi-
UA2S
40
30
20
10
0
-10
1
705
1409
2113
2817
3521
4225
4929
5633
6337
7041
7745
8449
9153
9857
10561
11265
11969
12673
13377
14081
14785

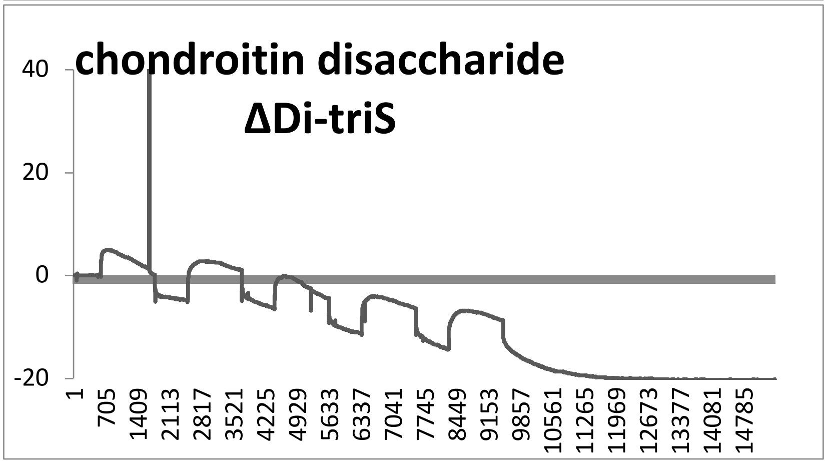
chondroitin disaccharide
ΔDi-triS
40
20
0
-20
1
705
1409
2113
2817
3521
4225
4929
5633
6337
7041
7745
8449
9153
9857
10561
11265
11969
12673
13377
14081
14785

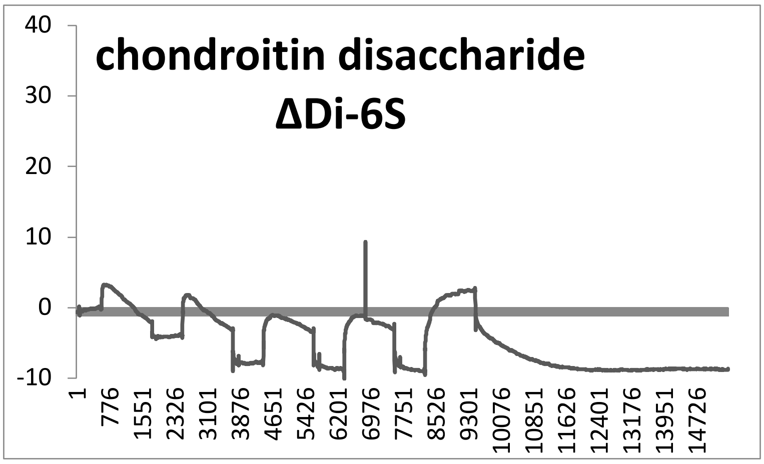
chondroitin disaccharide
ΔDi-6S
40
30
20
10
0
-10
1
776
1551
2326
3101
3876
4651
5426
6201
6976
7751
8526
9301
10076
10851
11626
12401
13176
13951
14726

Supplementary dataset S2. (A) Representative sensorgram from surface plasmon resonance (SPR) analysis of NHBA-DNA interactions. Graph shows response units (y-axis) over time in deciseconds (x- axis). (B) Agarose gel electrophoresis of 503bp PCR product used in SPR.

A

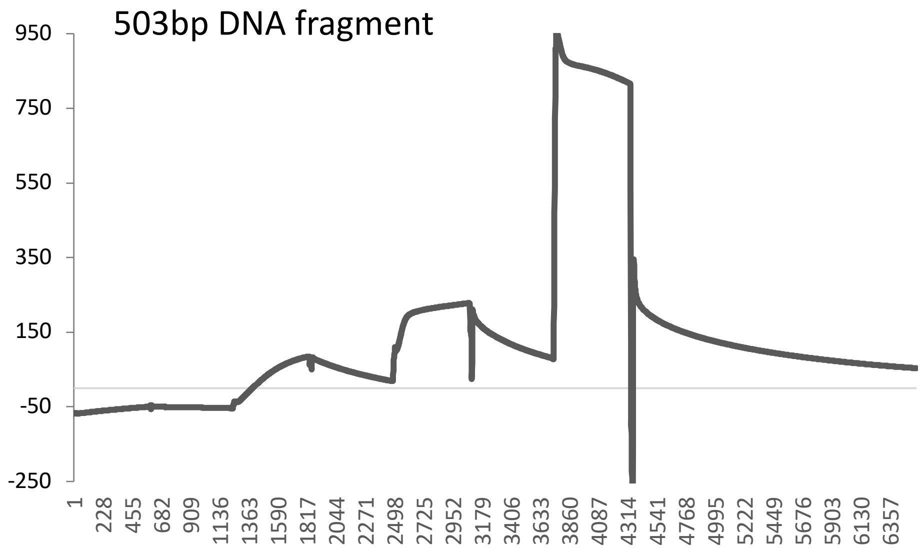

B

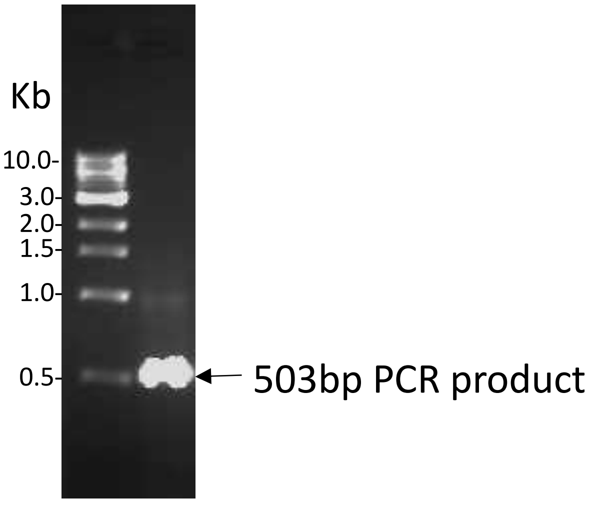

Supplement: Supplementary file 1 — Supplementary information [file 41598_2018_24639_MOESM1_ESM.pdf]
